# Kinase inhibitor library screening identifies synergistic drug combinations effective in sensitive and resistant melanoma cells

**DOI:** 10.1186/s13046-019-1038-x

**Published:** 2019-02-06

**Authors:** Christiane Margue, Demetra Philippidou, Ines Kozar, Giulia Cesi, Paul Felten, Dagmar Kulms, Elisabeth Letellier, Claude Haan, Stephanie Kreis

**Affiliations:** 1 Life Sciences Research Unit, University of Luxembourg, 6, av. du Swing, L-4367 Belvaux, Luxembourg; 2 Experimental Dermatology, Department of Dermatology, Technical University Dresden, Dresden, Germany

**Keywords:** Melanoma, Resistance, Kinase inhibitor, Cell cycle checkpoints, DNA damage response

## Abstract

**Background:**

Melanoma is the most aggressive and deadly form of skin cancer with increasing case numbers worldwide. The development of inhibitors targeting mutated BRAF (found in around 60% of melanoma patients) has markedly improved overall survival of patients with late-stage tumors, even more so when combined with MEK inhibitors targeting the same signaling pathway. However, invariably patients become resistant to this targeted therapy resulting in rapid progression with treatment-refractory disease. The purpose of this study was the identification of new kinase inhibitors that do not lead to the development of resistance in combination with BRAF inhibitors (BRAFi), or that could be of clinical benefit as a 2nd line treatment for late-stage melanoma patients that have already developed resistance.

**Methods:**

We have screened a 274-compound kinase inhibitor library in 3 BRAF mutant melanoma cell lines (each one sensitive or made resistant to 2 distinct BRAFi). The screening results were validated by dose-response studies and confirmed the killing efficacies of many kinase inhibitors. Two different tools were applied to investigate and quantify potential synergistic effects of drug combinations: the Chou-Talalay method and the Synergyfinder application. In order to exclude that resistance to the new treatments might occur at later time points, synergistic combinations were administered to fluorescently labelled parental and resistant cells over a period of > 10 weeks.

**Results:**

Eight inhibitors targeting Wee1, Checkpoint kinase 1/2, Aurora kinase, MEK, Polo-like kinase, PI3K and Focal adhesion kinase killed melanoma cells synergistically when combined with a BRAFi. Additionally, combination of a Wee1 and Chk inhibitor showed synergistic killing effects not only on sensitive cell lines, but also on intrinsically BRAFi- and treatment induced-resistant melanoma cells. First in vivo studies confirmed these observations. Interestingly, continuous treatment with several of these drugs, alone or in combination, did not lead to emergence of resistance.

**Conclusions:**

Here, we have identified new, previously unexplored (in the framework of BRAFi resistance) inhibitors that have an effect not only on sensitive but also on BRAFi-resistant cells. These promising combinations together with the new immunotherapies could be an important step towards improved 1st and 2nd line treatments for late-stage melanoma patients.

**Electronic supplementary material:**

The online version of this article (10.1186/s13046-019-1038-x) contains supplementary material, which is available to authorized users.

## Background

Melanoma is a very aggressive form of skin cancer where advanced stages are generally associated with poor patient survival [1]. Together with lung cancer, melanoma is characterized by the highest number of somatic mutations, mostly due to the exposure to environmental mutagens such as tobacco smoke or UV rays, respectively [2]. Mutations in the Ser/Thr-kinase BRAF (especially the V600E mutation) are responsible for abnormal MAPK pathway signaling in more than 50% of melanoma patients. The development of a targeted therapy against this constitutively active BRAF has revolutionized the treatment of late-stage melanoma patients. In 2011, two BRAF inhibitors (Vemurafenib and Dabrafenib) were approved by the Food and Drug Administration for clinical use in late-stage patients [3, 4]. However, the emergence of rapid drug resistance in most patients challenges the overall positive response rates for these inhibitors. Therefore, a combination therapy targeting BRAF together with MEK, a downstream kinase in the same signaling pathway (by using Trametinib or Cobimetinib) was approved in 2015 for stage III and IV melanoma patients [5] increasing the overall survival from approximatively 15 months for single treatments to 25 months and delaying the onset of drug resistance [6, 7]. Nevertheless, drug resistance also occurs after combined BRAF/MEK inhibition causing unresolved clinical issues. A number of mechanisms conferring kinase inhibitor resistance have been described [8], many of which converge on a process termed “phenotype switching”, whereby melanoma cells escape inhibition by reversibly adapting proliferation rates, by metabolic re-wiring, and by differentiation/de-differentiation processes.

Recently, immunotherapy with immune checkpoint inhibitors has become a promising 2nd line treatment for resistant patients or a 1st line treatment for BRAF wild-type (wt) patients, however, response rates are not exceeding 30% and treatment can be accompanied by severe side effects [9, 10].

Melanoma are very heterogeneous tumors [11, 12] and individual cells can gain additional mutations when evolving from primary lesions to metastasis or when subjected to therapy, which can be a major limitation to durable therapeutic responses. Therefore, the identification of novel drugs or drug combinations to delay or completely abolish the onset of resistance by targeting alternative pathways, proteins involved in cell cycle progression or the DNA damage response (DDR) pathway [13, 14], remains a clinically very important task and a number of new drugs are currently in clinical trials (reviewed in [15]).

Most intrinsic or acquired mutations (under treatment pressure) inevitably lead to the reactivation of the MAPK signaling pathway or activation of the PI3K/Akt pathways, all driving proliferation of cancer cells. As most signaling pathways involve kinases, we screened a kinase inhibitor library in parental melanoma cell lines alongside their corresponding Vemurafenib- and Dabrafenib-resistant sublines in order to identify new potential targets. We determined potent combinations of kinase inhibitors, which showed long-lasting synergistic effects in the treated cells.

## Methods

### Cell lines and cell culture

The melanoma cell line A375 was obtained from ATCC, IGR37 and IGR39 melanoma cells were purchased from DSMZ and 501Mel melanoma cells were acquired from Dr. Ruth Halaban (Dermatology department, Yale School of Medicine, USA). Primary BRAF mut melanoma M45 cells were freshly isolated from a patient muscle metastasis at the Dermatology department, Technical University Dresden in Germany. The chopped tumor was incubated in HBBS (w/o Ca^2+^ and Mg^2+^) containing 0.05% collagenase, 0.1% hyaluronidase, 1.25 U/ml dispase 20 mM HEPES, 100 g/ml gentamycin; 100 U/ml penicillin and 100 g/ml streptomycin, for 60 min at 37 °C. After centrifugation, cell pellets were washed in HBSS/20 mM HEPES and maintained in RPMI + 10% FCS. The use of patient material for biochemical analysis was approved by the ethics committee of the TU-Dresden (EK 65032013) and informed consent was obtained from the patient. The drug-resistant counterparts of A375, IGR37 and 501Mel cells (-XP: Vemurafenib (PLX4032)-resistant, -GP: Dabrafenib (GSK2118436)-resistant) were produced as described before [16]: Drug-resistant melanoma cell pools were generated from parental A375, IGR37, and 501Mel cells by long-term culturing under continuous presence of 5 μM Vemurafenib (PLX4032) or 100 nM Dabrafenib (GSK2118436). Inhibitor-containing media were exchanged three times a week. The parental A375 and resistant -XP and -GP derivatives were stably transduced with the near-infrared fluorescent protein (iRFP) using the LV-iRFP-P2A-Puro lentiviral particles (Imanis Life Sciences) as described in [17]. All cells were maintained in RPMI-1640 containing GlutaMAX™ (GIBCO) and supplemented with 10% FCS (GIBCO), 50 μg/ml penicillin (LONZA) and 100 μg/ml streptomycin (LONZA). The BRAFi-resistant cell lines and the iRFP-transduced cells were maintained under continuous presence of the inhibitor (Vemurafenib or Dabrafenib, Selleck Chemicals) and the selection antibiotic (Puromycin, Invivogen) respectively. Normal human epidermal melanocytes NHEM (PromoCell) were maintained in serum- and PMA-free MGM-M2 medium. Normal Human Dermal Fibroblasts, NHDF (PromoCell) were grown in DMEM containing GlutaMAX™ (GIBCO) and supplemented with 10% FCS (GIBCO), 50 μg/ml penicillin (LONZA) and 100 μg/ml streptomycin (LONZA). All cells were cultured in a humidified atmosphere with 5% CO_2_ supply and were regularly PCR-tested to be mycoplasma-negative.

### Kinase inhibitor library

The kinase inhibitor library, composed of 274 compounds dissolved in DMSO in a stock concentration of 10 mM, was purchased from Selleck Chemicals (Houston, USA). A375, IGR37 and 501Mel parental and BRAFi-resistant cells were seeded at a density of 0.5 × 10^4^ cells/well in 96-well black μclear plates (Greiner) in the presence of 1 or 10 μM inhibitor. After 72 h of treatment, cell viability was assessed using the PrestoBlue Cell Viability Reagent (ThermoFisher Scientific). Measurements were performed with the CLARIOstar Monochromator microplate reader (BMG Labtech).

### Dose-response curves and determination of IC_50_ values

All inhibitors used in this study were purchased from Selleck Chemicals (Houston, USA). They were dissolved in DMSO to a stock concentration of 10 mM according to the manufacturer’s instructions and stored at − 80 °C. Briefly, A375, IGR37 and 501Mel parental and their BRAFi-resistant -XP and-GP derivatives were seeded at a density of 0.5 × 10^4^ cells/well in 96-well black μclear plates (Greiner). Eight different dilutions (in a 3-fold dilution series) of each inhibitor (ranging from 0.05 to 10,000 nM) were assayed in technical triplicates for 72 h in each experiment. Cell viability was measured with the PrestoBlue Cell Viability Reagent (ThermoFisher Scientific) on a CLARIOstar Monochromator microplate reader (BMG Labtech). Using the GraphPad Prism v5.04 software, the half-maximal inhibitor concentration values (IC_50_) were determined from the curve using the nonlinear log (inhibitor) vs response-variable slope (four parameters) equation. IC_50_ values were only determined for compounds which inhibited growth by more than 50%. In addition, the IC_50_ values were only considered if the software gave unambiguous results and the R^2^ value was above 0.92. The IC_50_’s were determined for 3 biological replicates and are listed with their standard deviations.

### Synergy determination with the Chou-Talalay method

The Chou-Talalay method [18] to determine possible synergistic effects of selected kinase inhibitor combinations was used as described in [19]. A375, IGR37 and 501Mel parental and BRAFi-resistant cells were seeded at a density of 0.5 × 10^4^ cells/well in 96-well black μclear plates (Greiner). Cells were treated with either single inhibitors or combinations thereof at indicated amounts in technical triplicates. The amounts were pre-determined from each inhibitor’s IC_50_ value and inhibitors were assayed in a defined dilution series and at constant ratio when combined. Cell viability was measured after 72 h of treatment, with the PrestoBlue Cell Viability Reagent (ThermoFisher Scientific) on a CLARIOstar reader (BMG Labtech). Combination Index (CI) values showing either synergy (< 1) or antagonism (> 1) were calculated with the CompuSyn software (ComboSyn, Inc).

### Synergy determination with the SynergyFinder method

A375 parental and the BRAFi-resistant -XP and-GP derivatives were seeded at a density of 0.5 × 10^4^ cells/well in 96-well black μclear plates (Greiner) and were further treated as described above. Synergy scoring was determined using the “inhibition readout” (calculated as “100 - Cell Viability”) on the online SynergyFinder software (https://synergyfinder.fimm.fi) [20] and implementing the ZIP calculation method [21].

### Apoptosis assays

Apoptosis assays were performed by monitoring the caspase-3 activity through cleavage of the Ac-DEVD-AFC peptide (AlfaAesar) and the release of fluorogenic AFC (= 7-Amino-4-trifluoromethylcoumarin) in solution. Briefly, A375 parental and BRAFi-resistant cells were seeded at a density of 0.5 × 10^4^ cells/well in 96-well black μclear plates (Greiner). Cells were left untreated or were treated with 200 μM Etoposide (Sigma-Aldrich), used as a positive control of apoptosis, or MK-1775 and AZD7762 inhibitors either single or in combination at the indicated amounts and in technical triplicates. After 24 h of inhibitor treatment, cells were lysed for 30 min at 37 °C with 3x ReLy Buffer (150 mM Tris (pH 7.4), 300 mM NaCl, 30% glycerol, 1% Triton-X, 0.3% CHAPS, 6 mM EDTA (pH 8.0), 6 mM DTT, 75 μM Ac-DEVD-AFC) and free AFC was quantified on a CLARIOstar reader (BMG Labtech). To ensure specificity of the assay, caspase-3 mediated cleavage of the Ac-DEVD-AFC peptide was in parallel blocked by addition of the potent Ac-DEVD-CHO caspase-3 inhibitor (AlfaAesar) at a concentration of 25 μM. Statistical significance was determined with one-way repeated measures ANOVA followed by Dunnett’s post-test using the Graphpad Prism Software.

### Long-term inhibitor treatment

A375 parental and BRAFi-resistant cells, virally transduced with the near-infrared fluorescent protein (iRFP) were seeded at a density of 2500 cells/well in a 24-well plate (Greiner) in at least technical triplicates. Inhibitors, single or in combination, were added at the indicated amounts and replenished every 72 h for a duration of 76 days. To assess whether any remaining cells have become resistant to the treatment and would re-emerge, the drugs were removed and the cells underwent “drug holidays” for an additional 21 days. During the long-term treatment, cell proliferation was monitored bi-weekly on a LI-COR Odyssey Infrared Imaging System (LI-COR Biosciences). Fluorescence intensity was quantified with the Image Studio™ Lite software (LI-COR Biosciences).

### Western blots

Cells were seeded at a density of 10^5^ cells/well in 24-well plates (Greiner). 24 h post seeding, cells were treated with inhibitors at indicated amounts for 3 h and/or 24 h. Western blot analysis was performed as described before [22]. The following primary antibodies were used: anti-phospho-CHK1 (Ser317) (Cell Signalling Technology, 1:1000), anti-CHK1 (Cell Signalling Technology, 1:1000), anti-phospho-cdc2 (Tyr15) (Cell Signalling Technology, 1:1000), anti-cdc2 (Cell Signalling Technology, 1:1000), anti-PARP (Cell Signalling Technology, 1:1000), anti-cleaved-PARP (Cell Signalling Technology, 1:1000), anti-phospho-Erk (Tyr202/Tyr204) (Cell Signalling Technology, 1:2000), anti-Erk1/2 (Santa Cruz, Erk1 = 1:1000, Erk2 = 1:2000), anti-phospho-Akt (Ser473) (Cell Signalling Technology, 1:1500), anti-Akt1/2 (Santa Cruz, 1:1000), anti-Vinculin (Abcam, 1:1000) and anti-Tubulin (Santa Cruz, 1:5000). HRP-labeled secondary antibodies were purchased from Cell Signaling Technology (Boston, MA).

### In vivo assays

NOD scid gamma (NSG) mice were bred in-house and experiments were performed according to all applicable laws and regulations, after receiving approval by the institution’s animal experimentation ethics committee and the veterinarian service of the Ministry of Agriculture (Permit Number: 18-MDM-01). Single parental sensitive A375 cells (0.5 × 10^6^ cells) and Vemurafenib-resistant A375-XP cells (2 × 10^6^ cells) were resuspended in 100 μL of 1:1 mixed serum-free medium and matrigel (BD Biosciences) and injected subcutaneously in 6–8 week-old mice. At day 14, mice (*n* = 10 mice/group for A375 cells and *n* = 5 mice/group for A375-XP cells) had tumors with volumes of approximately 150mm^3^. Daily treatment was started for 8 consecutive days with vehicle, 40 mg/kg MK-1775 (formulated in 0.5% methylcellulose) given by oral gavage, 25 mg/kg AZD7762 (formulated in 11.3% 2-hydroxypropyl-β-cyclodextrin in 0.9% sterile saline) given by daily intraperitoneal injection, or the combination of MK-1775 and AZD7762. Control mice received the respective vehicle by oral gavage and intraperitoneal injection. Tumor growth was followed and tumor volumes were calculated by the formula 0.5236 × length × width × height. Statistical analysis was done using a two-way ANOVA followed by post-hoc Tukey’s multiple comparison tests.

## Results

### Kinase inhibitor library screening

**Fig. 1:**
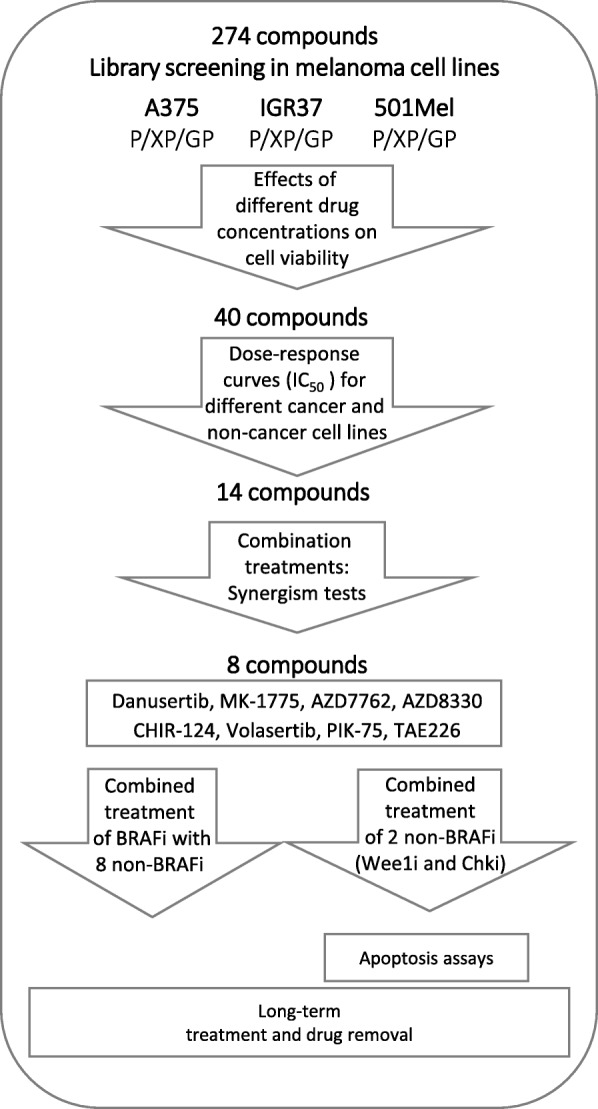
Study outline of kinase inhibitor screening. Three BRAF mutated melanoma cell lines, A375, IGR37 and 501Mel (each one in its parental (P), Vemurafenib (XP)- and Dabrafenib (GP)-resistant form) were treated with 2 concentrations (1 and 10 μM) of 274 different kinase inhibitors. 40 promising candidates were further characterized in dose-response assays, which led to the identification of 14 compounds that were used in combination treatments where synergism was assessed. Short- and long-term effects of combinations with 8 successful drugs were analyzed: Danusertib (Aurora kinase, FGFR, Bcr-Abl, c-RET, Src inhibitor), MK-1775 (Wee1 inhibitor), AZD7762 (Chk inhibitor), AZD8330 (MEK inhibitor), CHIR-124 (Chk inhibitor), Volasertib (Plk inhibitor), PIK-75 (PI3K, DNA-PK inhibitor), TAE226 (FAK inhibitor). BRAFi: BRAF inhibitor, Vemurafenib or Dabrafenib

In order to identify new kinases that could act as potential therapeutic targets to overcome BRAF inhibitor resistance, we screened a kinase inhibitor library of 274 compounds in 3 different melanoma cell lines that carry a mutated BRAF gene and are wild-type for NRAS: A375 cells with homozygous BRAF V600E and IGR37 and 501Mel cells heterozygous for BRAF V600E. All 3 cell lines were sensitive to the BRAF inhibitors Vemurafenib and Dabrafenib (Table 1 and [16]). Matching cells resistant to these 2 BRAF inhibitors had previously been generated and characterized in our lab [16]. The parental and 2 corresponding BRAFi-resistant cell lines were incubated for 72 h with 2 different concentrations (1 and 10 μM) of the individual drugs and cell viability was assessed (Additional file 1: Table S1). For further testing, we chose 40 inhibitors that either (i) had effects at low (1 μM) concentrations (e.g. Dinaciclib (CDKi), PIK-75 (PI3Ki, DNA-PKi), Trametinib (MEKi)), (ii) showed differences between parental and resistant cells (e.g. NVP-BHG712 (VEGFRi, Srci, Rafi, Bcr-Abli), Temsirolimus (mTORi), Sorafenib (VEGFRi, PDGFRi, Rafi)), (iii) had been described in literature to affect melanoma cells (e.g. MK-1775 (Wee1i), AZD7762 (Chki), Danusertib (AURKi, FGFRi, Bcr-Abli, c-RETi, Srci)) or iv) had comparable effects in at least 2 of the 3 different cell lines (e.g. ON-01910/Rigosertib (Plki), KX2–391 (Srci)). An overview of the study design and the selection of compounds for next round evaluations and combinatorial testing in short- and long-term experiments are shown in Fig. 1 and Table 1 respectively.

**Table 1:**
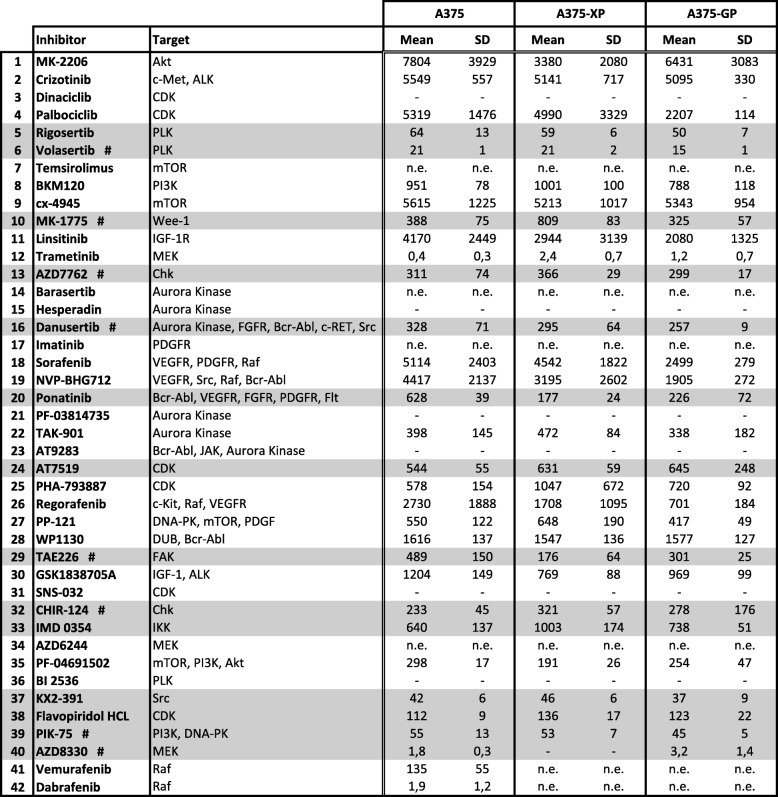
Half-maximal inhibitory concentration value (IC_50_) for selected kinase inhibitors

Parental and BRAFi-resistant A375 melanoma cells underwent treatment for 72 h with 3-fold serial dilutions of each kinase inhibitor and cell viability was assessed with the PrestoBlue Cell Viability assay. The IC_50_values (nM) were calculated as described in Methods. Values represent the mean of at least three biological replicates. Highlighted in grey are inhibitors chosen for synergy studies and marked additionally with a hash (#) are inhibitors chosen for long-term testing. “n.e.”: not efficient, inhibitors did not suppress growth below 50% in the tested concentration range. “-”: values could not be determined in GraphPad. SD: standard deviation; -XP: cells resistant to Vemurafenib, -GP: cells resistant to Dabrafenib

### Dose-response assays of 40 selected inhibitors on parental and resistant melanoma cells

To validate the effects of the 40 selected inhibitors, we generated dose-response curves by assessing cell viability and calculating IC_50_ values for A375 cells: parental and 2 corresponding resistant derivatives (labeled -XP when resistant to Vemurafenib and -GP when resistant to Dabrafenib [16]) (Additional files 2 and 3: Figure S1 and Table 1). To consolidate the data obtained in A375 cells, we further tested 20 of the chosen inhibitors in IGR37 and 501Mel cells (Fig. 3a and Additional file 4: Figure S2).

From these results, 14 inhibitors were selected based on their reproducibility, potency, and the quality of the curves obtained: we chose drugs that either potently inhibited proliferation in all 3 cell lines (Rigosertib, MK-1775, AZD7762) or that targeted pathways not directly linked to the MAPK pathway (IMD 0354 targeting IKK, or PIK-75 targeting PI3K and DNA-PK). Only drugs that presented acceptable dose response curves with IC_50_ values well below 1 μM were further analyzed. Overall, 8 inhibitors of kinases involved in cell cycle regulation (targeting cyclin-dependent kinases (CDKs), Aurora kinases (Aurks), Polo-like kinases (Plks), checkpoint kinases (Chks)), along with 6 inhibitors targeting different signaling pathways (see compounds marked in grey in Table 1) showed best results.

### Assessment of combinatorial effects of selected kinase inhibitors with BRAF inhibitors

**Fig. 2:**
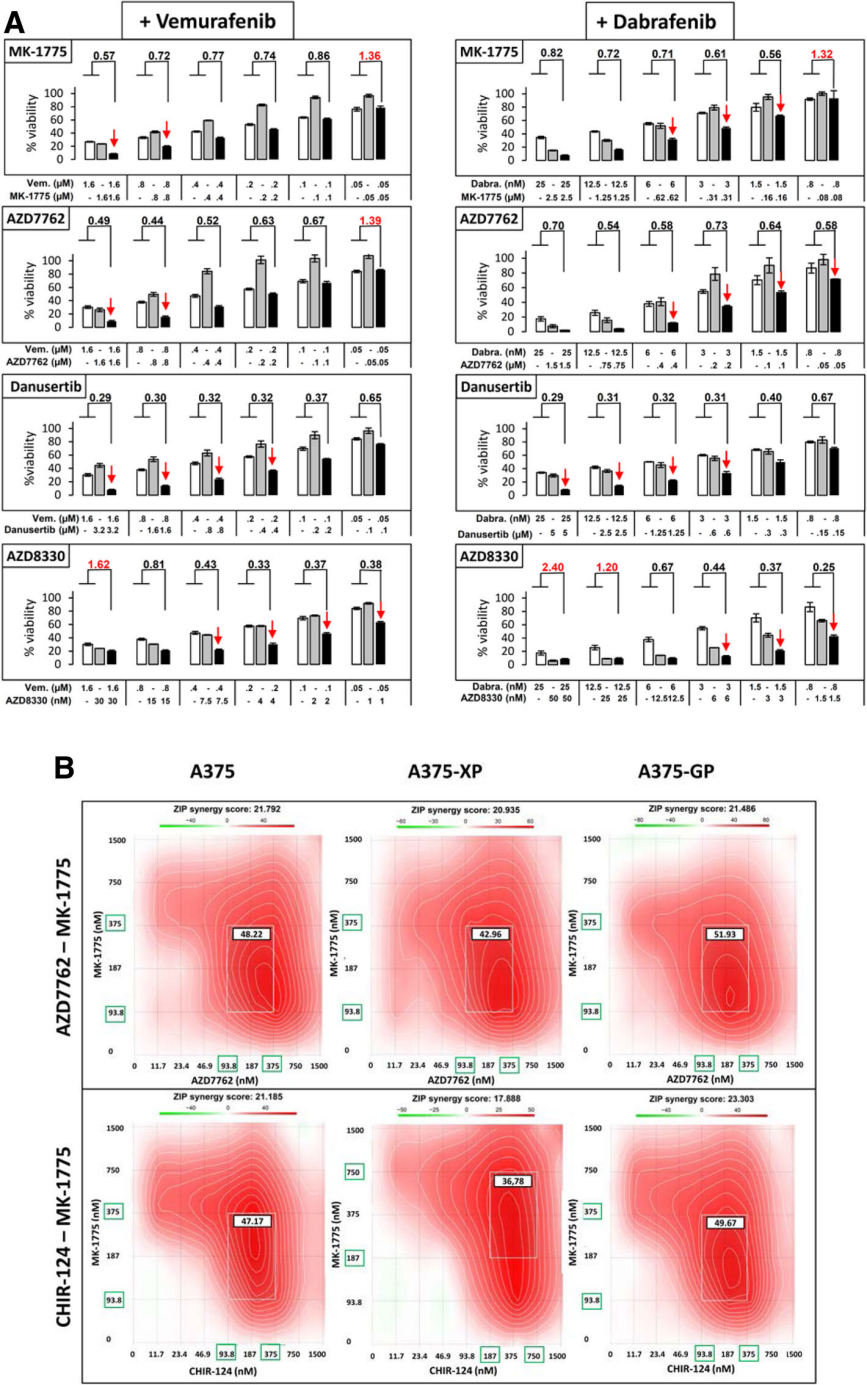
Combinations of different kinase inhibitors show synergistic effects in drug-sensitive and –resistant melanoma cells. **a** A375 cells were treated for 72 h with BRAF inhibitors Vemurafenib or Dabrafenib alone or in combination with MK-1775 (Wee1i), AZD7762 (Chki), Danusertib (Aurki) or AZD8330 (MEKi) and cell viability was assessed. A dose-effect analysis of the drug combinations to determine synergism/antagonism based on the Chou-Talalay method was performed using the Compusyn software. Combination index (CI) values shown above the bars were mostly < 1 indicating a synergistic effect of both drugs at the specific concentrations. CI values > 1 (marked in red), indicate antagonism; white bars show BRAFi treatment alone, grey bars show the tested kinase inhibitor alone and black bars show the combined drugs. Red arrows pinpoint the most effective combinations. One representative experiment of at least 3 is shown. **b** Chki and Wee1i act synergistically on parental and BRAFi-resistant A375 cells. Parental and resistant A375 cells (-XP: Vemurafenib-resistant; -GP: Dabrafenib-resistant) were treated for 72 h with the indicated concentrations of 2 Chki (AZD7762 or CHIR-124) and a Wee1i (MK-1775) and cell viability was assessed. Synergy scores were calculated using the Synergyfinder software. ZIP Synergy scores > 0 indicate synergism (red regions) and scores < 0 indicate antagonism (green regions). Concentrations marked with green boxes on the x and y-axis indicate the concentrations encompassing the region of highest synergy (indicated by the white rectangle). The value in the white box represents the averaged synergy score for the region of highest synergy. One representative experiment of at least 4 is shown

Six of the 14 selected inhibitors (Rigosertib, Flavopiridol HCl, AT7519, KX2–391, IMD0354 and Ponatinib) did not act synergistically with the BRAF inhibitors (data not shown). For the remaining 8 compounds, synergistic effects with the BRAF inhibitors Vemurafenib and/or Dabrafenib were scored in A375 cells (Fig. 2a and Additional file 5: Figure S3). Synergism can be calculated with different methods using effect-based strategies, like the Bliss independence or the HSA (Highest Single Agent) model or by dose-effect-based strategies, like the Loewe additivity model [23]. Here we applied 2 different tools to assess synergy: the Chou-Talalay method based on Loewe additivity [18] and Synergyfinder, scoring synergism using the 4 main models, HSA, Loewe, BLISS and ZIP (Zero Interaction Potency) [20]. When the BRAF inhibitors Vemurafenib and Dabrafenib were combined with drugs targeting the cell cycle like MK-1775 (Wee1i), AZD7762 (Chki) and Danusertib (Aurki), clear synergistic effects were observed as indicated by CI (combination index) values < 1 calculated by the Chou-Talalay method (Fig. 2a). CI values > 1 (marked in red) illustrate antagonism, as seen for the lowest concentrations of MK-1775 and AZD7762. As expected, the combination of BRAF and the MEK inhibitor AZD8330 also showed synergistic effects at low concentrations (Fig. 2a, lower 2 panels). Further combinations of inhibitors with synergistic effects are shown in Additional file 5: Figure S3A. Interestingly CHIR-124 (Chki), Volasertib (Plki) and PIK-75 (PI3Ki) had synergistic effects only with Dabrafenib. Vemurafenib showed synergism when combined with TAE226 (FAKi). Additional file 5: Figure S3B confirms the synergistic combinations observed with the Chou-Talalay method, using Synergyfinder, another tool for drug combination analysis: red regions with synergy scores > 1 indicate synergism (the regions of highest synergy are marked by a white frame), whereas the green regions indicate antagonism. Concentrations of drugs determining the regions of highest synergy were generally in the lower ranges, further strengthening their role as potential drug combination partners.

To confirm the inhibition of Wee1 kinase by MK-1775, Tyr15 phosphorylation levels of CDK1 (P-cdc2) were tested [24] while specificity of the Chk1 inhibitor AZD7762 was assessed by measuring induced phosphorylation of Chk1 [25, 26] (Additional file 6: Figure S4a). Both inhibitors performed with the expected level of specificity. The inhibition of Erk phosphorylation by Vemurafenib was confirmed as observed before [17] and the FAKi TAE226 worked by blocking P-Akt levels as expected [27].

Taken together, we identified 8 kinase inhibitors which had synergistic effects on cell growth when combined with Vemurafenib and/or Dabrafenib.

### Combinatorial effects of cell cycle checkpoint inhibitors

The combination of Wee1 and Chk inhibitors both targeting key enzymes of cell cycle control can kill melanoma cells independent of their BRAF mutation status [28, 29]. Here, both types of cell cycle inhibitors (AZD7762 and CHIR-124 (Chki) and MK-1775 (Wee1i)) acted synergistically with the BRAF inhibitors (Fig. 2a, Additional file 5: Figure S3B). Therefore, we investigated whether a combination of these drugs would also be effective in BRAF inhibitor-resistant cells, as this would be of high clinical relevance. Indeed, when the Wee1 inhibitor MK-1775 was combined with the Chk inhibitors AZD7762 or CHIR-124, highly synergistic effects were scored at the concentrations tested (Fig. 2b) both for the sensitive but importantly also for the BRAFi-resistant A375 cells (-XP: resistant to Vemurafenib, -GP: resistant to Dabrafenib). The same results were obtained with Chou-Talalay analysis, which indicated very low CI values (Additional file 7: Figure S5A), and only at the lowest concentrations CHIR-124 and MK-1775 lost their synergistic effects.

**Fig. 3:**
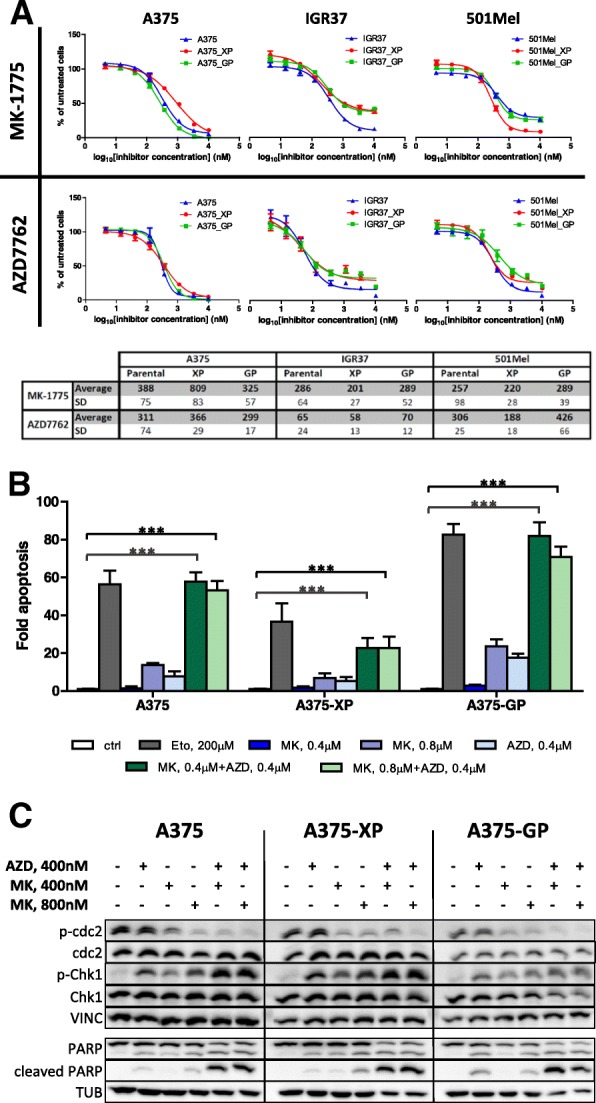
Effects of checkpoint inhibitors MK-1775 (Wee1i) and AZD7762 (Chki) on parental and resistant melanoma cells. **a** Dose-response curves and IC_50_ values (in nM) of MK-1775 and AZD7762 in A375, IGR37 and 501Mel melanoma cells. Response to 3-fold serial dilutions of each kinase inhibitor was assessed 72 h after treatment. The IC_50_ values (nM) were calculated as indicated in Methods. Values represent the mean of at least three biological replicates. SD: standard deviation; XP: cells resistant to Vemurafenib, GP: cells resistant to Dabrafenib. **b** The combination of MK-1775 and AZD7762 efficiently induces apoptosis in parental and BRAFi-resistant A375 cells. Cells were treated for 72 h with the indicated concentrations of MK-1775 (Wee1i) or AZD7762 (Chki) or a combination thereof. Etoposide (Eto) treatment was used as positive apoptosis control. Resulting caspase-3 activity was normalized to the untreated control. Error bars represent the standard deviation of four biological replicates. Statistical significance was determined with one-way repeated measures ANOVA followed by Dunnett’s post-test. *p > 0.05, **p > 0.01, ***p > 0.001. **c** Western blot analysis of A375, A375-XP and A375-GP cells after treatment for 3 or 24 h with indicated amounts of drugs. P-cdc2 (CDK1), cdc2 (CDK1), p-Chk1 and Chk1 were detected after 3 h drug treatment, while PARP cleavage was detected after 24 h treatment. Vinculin and α-tubulin were used as loading controls. AZD: AZD7762, MK: MK-1775

MK-1775-driven Wee1 inhibition in parental and resistant A375 cells reduced levels of inhibitory CDK1 (cdc2) phosphorylation, and this effect was enhanced by the Chki AZD7762 (Fig. 3c). Vice versa, when cells were treated with AZD7762, we observed increased levels of inhibitory Ser317 phosphorylation in Chk1, which were even more pronounced when Wee1 was inhibited in parallel (Fig. 3c). Similar effects were also observed in primary melanoma cells derived from a patient’s muscle metastasis (M45) (Additional file 6: Figure S4C).

To further consolidate these observations, the same treatments (MK-1775 combined with AZD7762) were applied to 2 additional melanoma cell lines, both sensitive and resistant to BRAF inhibitors, namely IGR37 and 501Mel, with identical results (Fig. 3a, Additional file 7: Figure S5B). An intrinsically resistant cell line to BRAFi, IGR39, was also tested and again we observed synergistic killing effects of the MK-1775/AZD7762 combination (Additional file 7: Figure S5C).

**Fig. 4:**
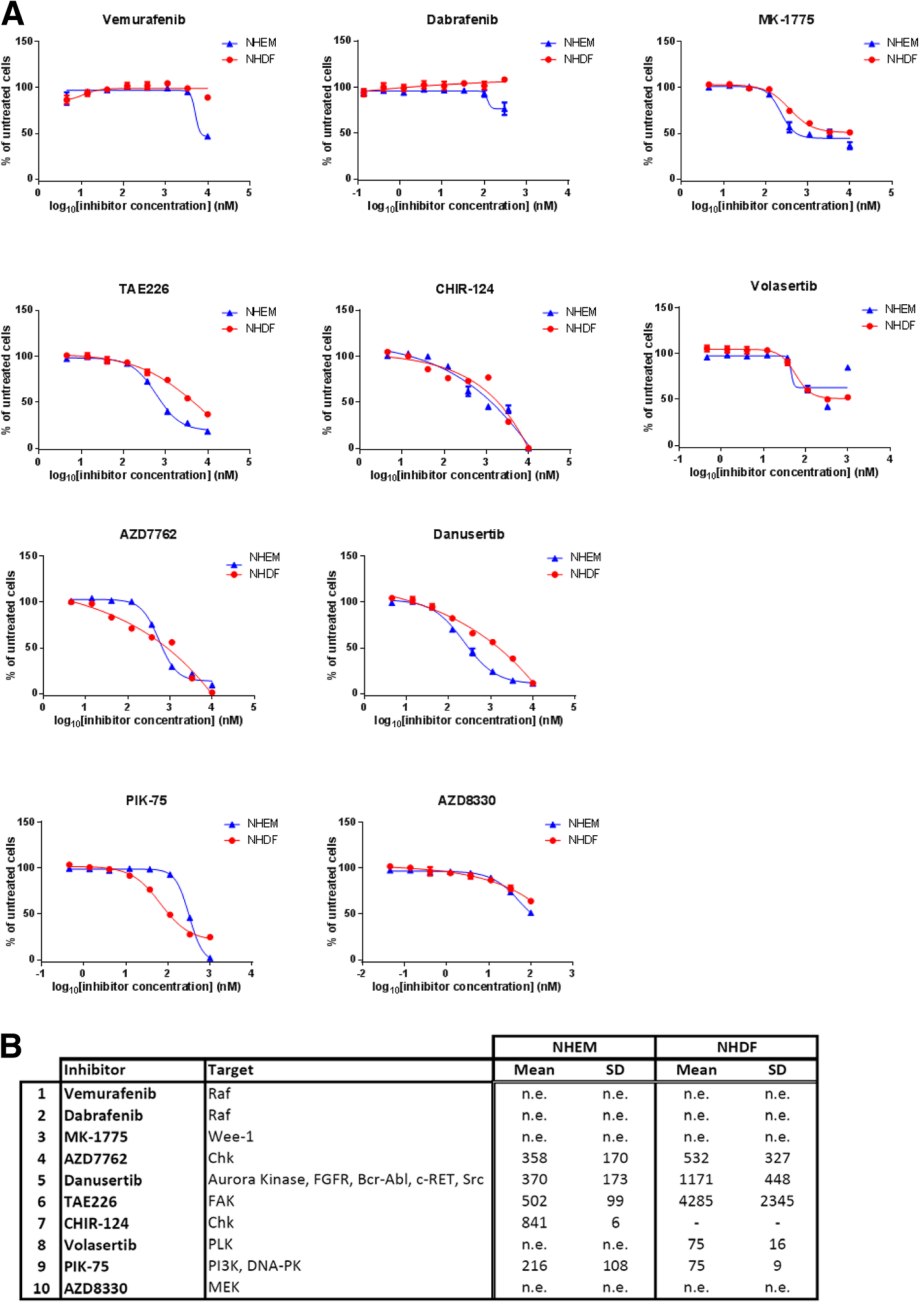
Dose-response studies of selected kinase inhibitors in non-cancer NHEM and NHDF cells. **a** Response to 3-fold serial dilutions of each kinase inhibitor was assessed 72 h after treatment by measuring cell viability. **b** The table indicates the half-maximal inhibitory concentrations (IC_50_) for the selected inhibitors. The IC_50_ values (nM) were calculated as indicated in Methods. Values represent the mean of at least three biological replicates. “n.e.”: not efficient: inhibitors did not suppress growth below 50% in the tested concentration range. “-”: values could not be determined in GraphPad. NHEM: normal human epidermal melanocytes; NHDF: normal human dermal fibroblasts

Next, we investigated if the combination of Wee1 and Chk inhibitors induces apoptosis rather than slowing down proliferation of parental and resistant A375 cells and of the primary M45 melanoma cells (Fig. 3b and c, Additional file 6: Figure S4). While single treatments with 400 or 800 nM Wee1 inhibitor (MK-1775) and 400 nM Chk inhibitor (AZD7762) (blue bars) increased apoptosis rates (measured by caspase-3 activity) up to only 20%, the combined application of both drugs (green bars) induced very high apoptosis levels similar to the Etoposide treatment (positive control) in parental and Dabrafenib-resistant cells, with less pronounced responses in Vemurafenib-resistant cells (Fig. 3b). Increasing the concentration of MK-1775 (800 nM) in the combination treatment did not enhance the effect further (light green bars). In general, A375 cells resistant to Dabrafenib (A375-GP) were more sensitive to apoptosis induction, either by the control treatment (Etoposide) or by the kinase inhibitors. Comparable results could also be detected in the M45 primary cells (Additional file 6: Figure S4B). Cleavage of PARP, another indicator for apoptosis, was also induced by treatment with either AZD7762, MK-1775 and even more so with their combination (Fig. 3c, Additional file 6: Figure S4C). Moreover, we tested the effects of candidate drugs on normal/healthy cells present in and around the tumor, namely melanocytes (NHEM) and fibroblasts (NHDF). As expected, the very specific BRAF inhibitors Vemurafenib and Dabrafenib did not have any effect on the healthy counterpart cells, alone or in combination with the different other inhibitors (Fig. 4 and Additional file 8: Figure S6). The Wee1 inhibitor MK-1775 and the Chki AZD7762 had very modest effects on healthy fibroblasts and melanocytes at lower concentrations, which is in line with results observed by Magnussen and colleagues [30]. When both drugs were combined, we observed nearly no caspase-3 activation and no PARP cleavage at all (Additional file 6: Figure S4).

**Fig. 5:**
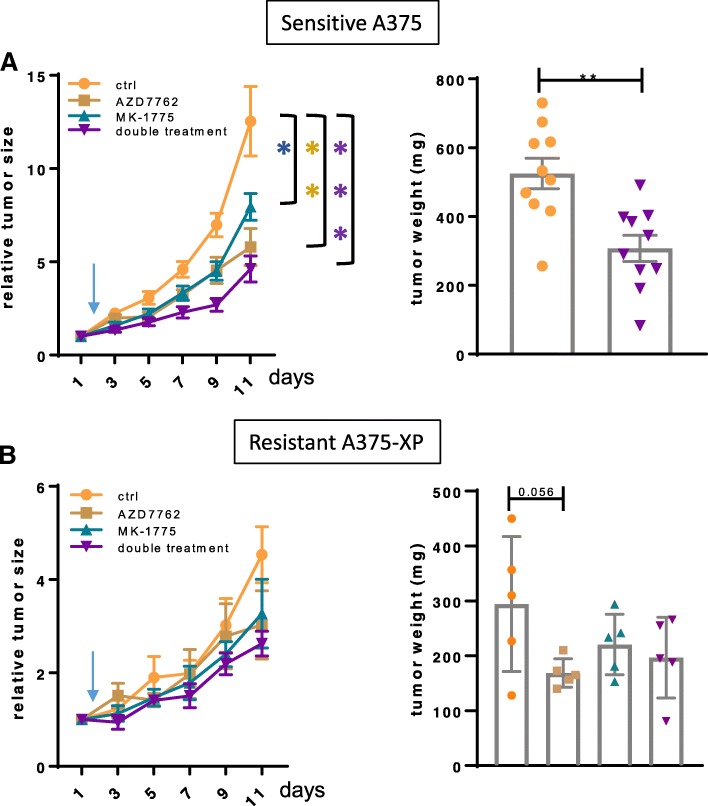
Combination treatments efficiently reduce growth of A375-derived melanoma tumors in mice. NSG mice were injected subcutaneously with A375 cells (**a**) (*n* = 10 mice/group) and A375-XP cells (**b**) (*n* = 5 mice/group). After 14 days, when tumors were approximately 150 mm^3^, treatment was initiated (indicated by an arrow) by daily gavage (MK-1775, Wee1 inhibitor) or daily intraperitoneal injection (AZD7762, Chk inhibitor) or a combination of both inhibitors, for 8 consecutive days. Tumor growth was evaluated every second day and weights of extracted tumors were measured at end-point. The tumor volumes are presented relative to the tumor volume at the day before initiation of treatment. Data are presented as means ± SEM, *p < 0.05, **p < 0.01 ***p < 0.001 compared to vehicle-treated tumors. Comparison of tumor volumes was performed with a two-way ANOVA between groups followed by post-hoc Tukey’s multiple comparison tests

The beneficial effects of combining Chk and Wee1 inhibitors have been demonstrated in BRAF mutant and wild-type melanoma cells, as well as in other human tumor cell lines [28, 29, 31, 32]. Here, we confirm these observations and additionally, our study highlights the efficacy of this combination especially on melanoma cells rather than healthy surrounding cells, and also importantly in the context of acquired and intrinsic BRAF inhibitor resistance. To assess the effect of Wee1 and Chk inhibition in vivo, we tested MK-1775, AZD7762 and the combination of both in mice injected subcutaneously with parental sensitive A375 cells or Vemurafenib-resistant A375-XP cells (Fig. 5). The combined inhibition of Wee1 and Chk stopped tumor growth in resistant and even more so in sensitive tumors, supporting the clinical relevance of our findings. Additionally, mice did not lose weight nor did they show any obvious side effects during treatment, altogether indicating no major toxicity issues.

### Long-term treatments

As patients are generally treated over longer periods of time, long-term in vitro studies are necessary to finally conclude on drug effects. The often used 48-72 h assays are not adequate to exclude that resistance will eventually occur, even with a combination of drugs that are synergistic using the above shown conditions.

Based on the promising synergistic effects (Fig. 2, Additional file 5: Figure S3 and Additional file 7: Figure S5), the following 8 kinase inhibitors were selected for further characterization in long-term experiments: Danusertib (Aurora kinase, FGFR, Bcr-Abl, c-RET, Src inhibitor), MK-1775 (Wee1i), AZD7762 (Chki), CHIR-124 (Chki), Volasertib (Plki), PIK-75 (PI3K, DNA-PK inhibitor) and TAE226 (FAKi) (see Table 1, marked in grey with #). We generated parental and resistant A375 cells stably expressing iRFP (near-infrared fluorescent protein) to be able to monitor effects of the inhibitors over time. iRFP fluorescence was quantified on a LICOR imaging system, allowing for continuous proliferation measurements following single kinase inhibitor or combined treatments. After 11 weeks in the presence of drugs, cells were fed for another 3 weeks with regular medium, in order to evaluate if combination treatments had killed all cells or if residual melanoma cells would resume their growth once the drugs were removed.

**Fig. 6:**
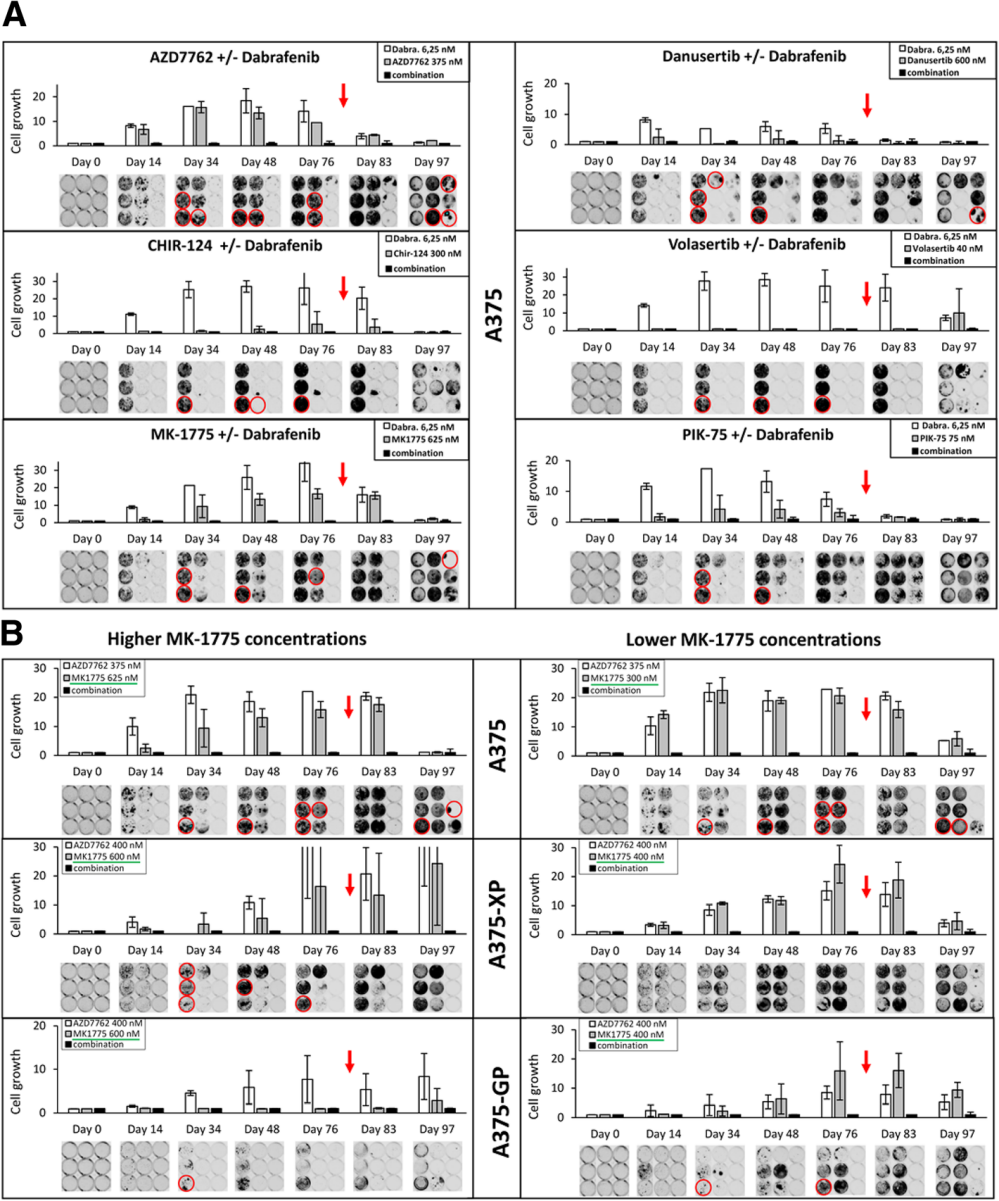
Long-term treatment of A375 cells with single inhibitors or synergistic drug combinations. **a** Parental A375 cells expressing iRFP were treated for up to 76 days (11 weeks) with the BRAF inhibitor Dabrafenib alone or in combination with selected kinase inhibitors. **b** Parental and BRAFi-resistant A375 cells (-XP: Vemurafenib-resistant; -GP: Dabrafenib-resistant) expressing iRFP were long-term treated with a Wee1i (MK-1775) alone or in combination with a Chki (AZD7762) at the indicated concentrations. Cell growth was monitored over time by quantifying fluorescence on a LICOR Odyssey Imaging system (at least 3 wells per treatment). Red circles indicate wells with saturation, where no fluorescence quantification in the linear range was possible. Red arrows mark the timepoint (after day 76) when drugs were removed from the medium. The combined treatment was set to 1. Dabra: Dabrafenib

In a first set of treatments, different kinase inhibitors were combined with Dabrafenib or Vemurafenib (data not shown) in parental A375 cells. Figure 6a clearly indicates that until day 76 (11 weeks, after this timepoint the drugs were removed) a combination of Dabrafenib with AZD7762 (Chki) or MK-1775 (Wee1i) was able to stop cell growth. As expected, cells had become resistant to the single treatments and continued to proliferate, except in presence of 300 nM CHIR-124 (another Chki) which killed most of the cells, opposed to 150 nM of this drug, which was not sufficient to prevent cells to grow back even in the presence of both drugs (with Dabrafenib, data not shown). This highlights the importance of treatment with adequate drug concentrations for prevention of tumor recurrence. PIK-75 together with Dabrafenib was also efficient in suppressing growth over extended periods of time, as compared to the single treatments. Regarding Danusertib, the results were inconsistent, with only 1 out of 3 wells showing resistant cells in the single treatment. A concentration of 40 nM Volasertib (Plki) (and also 20 nM, data not shown) killed all cells.

Notably, removal of most drugs from the cell medium after day 76 (marked by the red arrow) allowed for remaining cells to grow back even in the combined treatments. By day 97 (after 20 days without drugs) cells had re-grown for most treatments. The combined inhibition of BRAF and Plk (by Volasertib) marked an exception where hardly any surviving cells were detected at day 97, while cells treated with Volasertib alone had started to grow back. Identical results were obtained with 20 nM Volasertib (data not shown). Taken together, treatments combining BRAF inhibition with Chk (AZD7762, CHIR-124), Wee1 (MK-1775) or Plk (Volasertib) inhibition led to a more efficient response than with single treatments, although cells partially re-grew after removal of the drugs. Overall, these findings suggest that such combined treatments with optimized concentrations and treatment duration could have more durable clinical effects for advanced-stage melanoma patients. Ultimately, ideal concentrations and durations of single or combined drugs and their treatments have to be established in clinical trials.

Finally, we combined the most promising Chk and Wee1 inhibitors AZD7762 and MK-1775, since they showed high degrees of synergism in parental but also in several BRAF inhibitor-resistant melanoma cell lines, over longer periods of time. In parental A375 cells, a clear advantage of combinations, with no cell growth visible until day76 (with low and higher concentrations of MK-1775, Fig. 6b) was observed. After removal of drugs, at least 1 out of 3 wells of parental A375 cells showed few surviving cells.

Interestingly, cells resistant against either Vemurafenib or Dabrafenib, reacted differently to secondary drugs under short- and long-term exposure (Figs. 3 and 6). These findings argue for personalized 2nd line treatments, which may vary depending on the administered 1st line drug. Furthermore, long-term experiments illustrate that drugs (even in combination) that seem to act synergistically in 72 h assays can still induce resistance over longer exposure times, making them less likely to work in patients where drugs are usually administered over many months. Additionally, we could show that the combination of the Chk and Wee1 inhibitors is promising when treating parental, but also BRAF inhibitor resistant cells.

## Discussion

Even though melanoma has been the posterchild when it comes to advances in cancer treatment over the last few years, there is still a long way to go until a larger percentage of advanced stage patients can expect a much prolonged progression-free survival (PFS) or even to be cured. Largely, melanoma patients fall into 2 groups: BRAF wt and BRAF mutant, for which efficient targeted therapies exist. Combined BRAF and MEK inhibition have increased median PFS to 9–11 months [7], but could have much better clinical outcomes if it was not for the inevitable emergence of drug resistance. For BRAF wt (~ 50%) and also for drug-resistant patients, a new treatment option has become available in recent years: immunotherapy with PD-1 (Programmed cell death protein 1), PD-L1 (Programmed death-ligand 1) and CTLA4 (cytotoxic T-lymphocyte associated protein 4) immune checkpoint inhibitors.

Kinases represent one of the largest groups of druggable proteins: the 518 kinases encoded within the human genome are involved in basically all signaling pathways and their functions are often aberrantly regulated not only in cancer but also in immunological, metabolic or degenerative disorders. As a consequence, many successful kinase inhibitors have been developed for the clinical treatment of several cancers and other diseases (e.g. Imatinib (Gleevec), Crizotinib, Tofacitinib, Ruxolitinib). Currently, 43 inhibitors have FDA approval (www.brimr.org/PKI/PKIs.htm) [33]. The biggest part of these drugs are ATP competitive inhibitors, targeting the ATP pocket of the kinase either in its active (Type I inhibitors) or inactive (Type II inhibitors) state. Type III and IV inhibitors bind to pockets that are specific for each kinase, making them much more selective. To date, only 3 inhibitors (all of them MEK inhibitors: Trametinib, Cobimetinib and Binimetinib) are of the highly selective Type III (www.brimr.org/PKI/PKIs.htm) [33]. The ATP-binding site is highly conserved among all kinases, and for this reason the so called “poly-pharmacology effect” (inhibitors targeting more than one protein) is often observed with Type I and II inhibitors. These off-target effects can have detrimental consequences (severe side effects of treatment) for patients, however they could also be exploited for drug repurposing. Recent studies have profiled kinase inhibitors across the kinome [33–36] to get further insights into their target specificity and potency, their kinase and non-kinase off-target effects [37].

**Fig. 7:**
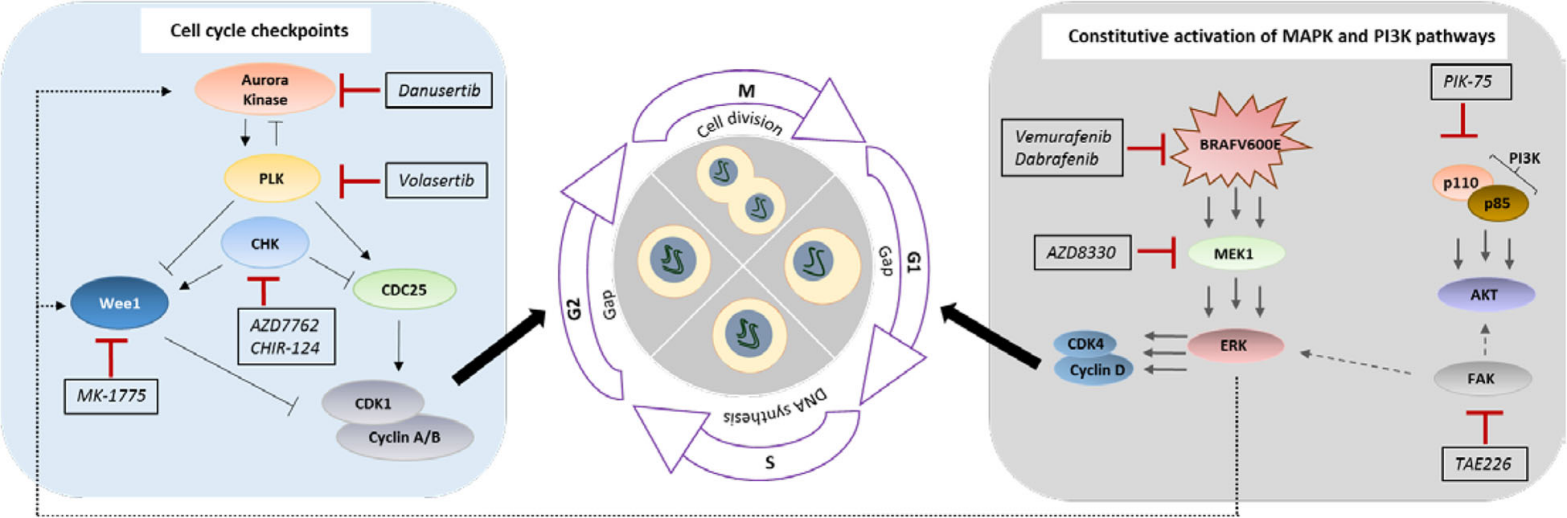
Pathways affected by the combined selected kinase inhibitors in BRAF mutant melanoma cells. Scheme summarizing the effects of cell cycle-influencing proteins targeted by the identified kinase inhibitors (black boxes). The pathway on the right (grey background) represents mitogenic factor-induced or mutated BRAF-induced entry into the cell cycle, while the left box (blue background) depicts players involved in progression through the different checkpoints and phases of the cell cycle. cdc25: cell division cycle 25

In order to find new combinations of kinase inhibitors to treat BRAFi-resistant melanoma, delay or avoid development of resistance, we have screened a 274-kinase inhibitor library in BRAF mutant melanoma cell lines, sensitive and resistant to different BRAF inhibitors. Not surprisingly, the most potent responses were seen with inhibitors targeting cell cycle regulators, which have strong effects on cell growth, already when given on their own [14, 38]. Important players include the cyclin-dependent kinases (CDKs), the DNA damage checkpoint kinases Chk1, Chk2 and Wee1, and the mitotic spindle assembly checkpoint Polo-like kinases (Plk) as well as Aurora kinases (Aurk) (see Fig. 7). Numerous inhibitors targeting these kinases are available, with some already in clinical use (e.g. the highly specific CDK4 and − 6 inhibitors Palbociclib and Ribociclib for breast cancer patients). Different rationales speak for targeting cell cycle proteins:

(i) Aberrantly regulated CDKs (in melanoma 75 to 90% of tumors show mutations in the p16INK4A-cyclinD-CDK4/6-Rb pathway) (reviewed in [39]) allow for uncontrolled tumor growth overriding crucially important checkpoints, making these kinases very prominent drug targets. Many CDK inhibitors are currently in clinical trials for melanoma treatment [39, 40].
(ii) Aiming at checkpoint kinases like Chk1 or Wee1 that normally halt cell cycle progression to allow for DNA damage repair would prevent these arrests and drive cell proliferation, despite the accumulation of DNA damage, possibly leading to apoptosis during mitosis (“mitotic catastrophe”) [41]. Replicative stress (RS), which is the interruption of replication fork progression and/or DNA synthesis during replication, can be induced by depletion of nucleotide pools, reactive oxygen species (ROS), oncogenic signaling and tumor suppressor inactivation, all present in melanoma cells. Considerable RS, an uncommon feature of normal cells, can lead to apoptotic and non-apoptotic cell death [13, 42]. RS is also a strong activator of Chk1 and Wee1, again making these checkpoint proteins interesting drug candidates. So far, the Wee1 inhibitor MK-1775 (AZD-1775) is being tested in clinical studies, alone and in combination with multiple other compounds. At present (July 2018) 50 studies for MK-1775 were recorded in ClinicalTrials.gov, only 1 of them including melanoma patients. Also several Chk1 inhibitors have been tested, among them AZD7762, that had to be discontinued due to high cardiac toxicity [43].
(iii) Like anti-mitotic drugs (e.g. Taxol), the therapeutic targeting of the mitotic Polo-like (Plk) and Aurora kinases, overexpressed in many cancer types (reviewed in [14, 44]), can induce mitotic cell arrest and cell death. Volasertib and Rigosertib targeting Plk1 are 2 promising inhibitors inducing cell cycle arrest and apoptosis, which are in clinical trials for different cancer entities, except melanoma. Likewise, various inhibitors of the A and B members of the Aurora kinase family (e.g. Alisertib, Danusertib) are in clinical studies [14].

One of the goals of our study was to find novel combination treatments with BRAF inhibitors that would act synergistically and could prevent or overcome resistance. We have identified several drugs that target the DNA damage checkpoint kinases Chk1/2 and Wee1 (namely AZD7762, CHIR-124 and MK-1775, respectively) and that work synergistically with BRAF inhibitors, as quantified through an effect based (Synergyfinder) and a dose-effect based (Chou-Talalay) methodology (Fig. 2a and Additional file 5: Figure S3). Interestingly, Wee1 kinase was identified as a downstream target of BRAF V600E [45] and its expression was found to be upregulated in melanoma as compared to benign nevi [30]. This combined inhibition of mutated BRAF and Wee1 is a very good example of vertical pathway inhibition where 2 players within the same oncogenic pathway are targeted, making the treatment more efficacious and lowering the chances of resistance development. In our hands, even after 11 weeks of combined treatment with Dabrafenib and MK-1775, cells had not become resistant (Fig. 6a). A few surviving cells were able to grow back after the drugs had been removed from the medium, indicating the need for further adjustments of initial drug concentrations and duration of treatment. The same observations were made when BRAF inhibitors were combined with the Chk inhibitors AZD7762 and CHIR-124.

Targeting the Aurora kinases A and B with Danusertib in combination with the BRAF inhibitors Vemurafenib and Dabrafenib synergistically killed A375 melanoma cells (Fig. 2a). This pan-Aurora kinase inhibitor also induced apoptosis and inhibited migration of several other melanoma cell lines [46].

Volasertib, an inhibitor of the mitotic kinase Plk1, had strong synergistic effects with the BRAF inhibitor Dabrafenib (Additional file 5: Figure S3A). Long-term experiments showed that 40 nM of Volasertib on its own was enough to keep cells in check, but when inhibitors were removed from the medium after 11 weeks, only the combined treatment with Dabrafenib and Volasertib was able to prevent recurrence of cell growth. Volasertib (BI 6727) had been shown to delay growth of melanoma tumors and to cause regression by inducing apoptosis in vivo [47] and, importantly, it showed synergistic antitumor effects together with the MEK inhibitor Trametinib in NRAS mutant melanoma [48]. This inhibitor has been studied extensively in over 25 clinical trials, but has not received FDA approval yet.

Furthermore, we have also observed synergistic effects of horizontal inhibition of the cross-talking pathways, PI3K/Akt and MAPK (Additional file 5: Figure S3A). A draw-back of this strategy is the appearance of dose-limiting toxicities, but many combinations are currently tested in clinical trials (reviewed in [49]).

Another interesting observation is the synergistic combination of Vemurafenib with the FAK inhibitor TAE226 (Additional file 5: Figure S3). FAK was recently described to be involved in the emergence of dedifferentiated, BRAFi-adapted cells and its inhibition increased killing effects of BRAF and MEK inhibitors [50]. Using a computational approach, Gayvert and colleagues discovered a synergistic interaction between Vemurafenib and FAK inhibitor 14 [51]. Very importantly, the tumor microenvironment, namely melanoma associated fibroblasts, were activated by BRAF inhibition inducing FAK-dependent melanoma survival and a combination of Vemurafenib with a FAK inhibitor lead to tumor regression in mouse allografts and patient-derived xenografts [52]. Notably, all of the above-mentioned inhibitors that had synergistic effects with BRAF inhibitors also very efficiently (low IC_50_ values) killed BRAFi-resistant melanoma cells (A375, IGR37, 501Mel) on their own (See Table 1, Fig. 3a, Additional file 3: Figure S1 and Additional file 4: Figure S2).

The combined inhibition of Wee1 and Chk1/2 has shown very promising effects when applied to other cancer cells but also melanoma cells (reviewed in [13]). Since we were interested in finding drug combinations that would also kill BRAFi-resistant melanoma cells, we extensively studied these drug pairs (AZD7762 (Chki) and MK-1775 (Wee1i) or CHIR124 (Chki) and MK-1775 (Wee1i)) in drug-naïve and -resistant melanoma cell lines (Figs. 2b, 5 and Additional file 7: Figure S5). We could clearly show that combined inhibition of Chk1/2 and Wee1 synergistically killed BRAFi-resistant cells and more importantly, reduced tumor growth by up to 50% in an in vivo melanoma mouse model. In long-term treatments over 11 weeks especially the BRAFi-resistant A375 cells (A375-XP and –GP) showed no or very little regrowth of cells when drugs were removed from the medium (Fig. 6b). These results indicate that the Wee1/Chk1/2 inhibition could be an encouraging therapeutic option for BRAFi-resistant melanoma patients.

Until entirely new treatment regimens such as more efficient immunotherapies, combinatorial inhibition with drugs targeting histone deacetylases (HDACs) or inducing apoptosis (reviewed in [15]) or immunotherapies together with oncolytic viruses [53, 54] as well as personalized cocktails of combined treatments come into practice, more efficient combinations of available and FDA-approved kinase inhibitors could improve progression-free survival of melanoma patients, who are not eligible to BRAF inhibition or have become resistant to this treatment. For those patients (at least 50%) other kinase inhibitor combinations, either given as targeted therapy alone or before or after immunotherapy, could become the treatment of choice.

## Conclusion

Taken together, we have identified and characterized synergistic kinase inhibitor treatments targeting the MAPK pathway and the cell cycle that could be promising alternatives for drug-resistant melanoma patients or wild-type BRAF patients. Interestingly, different drug combinations were effective depending on which one of two standard BRAF inhibitors was used in 1st line therapy. Finally, we show that fine-tuned drug concentrations and optimized treatment durations are necessary to achieve long lasting effects without rapid emergence of resistance.

## Additional files

Additional file 1: **Table S1.** Kinase inhibitor library screening. A commercial kinase inhibitor library of 274 compounds was tested on 3 different BRAF mutant melanoma cell lines, A375, IGR37 and 501Mel. Each cell line was treated in its sensitive form (parental), and also as 2 resistant derivatives, which were made resistant to either Vemurafenib (XP) or Dabrafenib (GP). Drugs were added at 2 different concentrations, 1 and 10 μM, for 72 h and cell viability was assessed using the PrestoBlue Cell Viability assay. Results are depicted as % viability compared to untreated cells. Each cell line was analyzed at least in duplicate. Inhibitors that were further analyzed and characterized in more detail are marked in red. (XLSX 95 kb)

Additional file 2: **Table S2.** Mutation status of genes related to this study. Data are based on own sequencing experiments (WES and Sanger sequencing), online available data from the “COSMIC cell lines project” (https://cancer.sanger.ac.uk/cell_lines) and on literature (Halaban et al., Pigm Cell Mel Res, 2010). Synonymous mutations or mutations in non-coding sequences were not taken into account here. wt: no mutation detected; ni: no information available. Genomic profiles (exome sequencing) of the cell lines (A375, -XP and –GP, IGR37, -XP and –GP and IGR39) are available upon request. (PDF 63 kb)

Additional file 3: **Figure S1.** Dose-response curves of selected kinase inhibitors in parental and BRAFi-resistant A375 cells. Response to 3-fold serial dilutions of each kinase inhibitor was assessed 72 h after treatment by measuring cell viability. Interesting candidates further tested in combination treatments in A375 cells are highlighted by a red frame (see also Table 1). One representative curve of at least 3 biological replicates is depicted here. _XP: cells resistant to Vemurafenib, _GP: cells resistant to Dabrafenib. (PDF 1030 kb)

Additional file 4: **Figure S2.** Dose-response curves of selected kinase inhibitors in parental and BRAFi-resistant IGR37 and 501Mel cells. Response to 3-fold serial dilutions of each kinase inhibitor was assessed 72 h after treatment by measuring cell viability in IGR37 (A) and 501Mel (B) cells. The values depicted in the different graphs indicate the half-maximal inhibitory concentrations (IC_50_) of inhibitors for which IC_50_ values could be determined (as explained in Methods). Values represent the mean of at least three biological replicates; one representative curve of at least 3 biological replicates is depicted. _XP: cells resistant to Vemurafenib (red), _GP: cells resistant to Dabrafenib (green). (PDF 304 kb)

Additional file 5: **Figure S3.** BRAF inhibitors in combination with selected kinase inhibitors synergistically inhibit proliferation of A375 melanoma cells. A) A375 cells were treated for 72 h with Dabrafenib alone or in combination with CHIR-124 (Chki), Volasertib (Plki) or PIK-75 (PI3Ki, DNA-PKi), or with Vemurafenib alone or combined with TAE226 (FAKi) and cell viability was determined . A dose-effect analysis of the drug combination based on the Chou-Talalay method was performed using the Compusyn software. CI values shown above the bars were mostly < 1 indicating a synergistic effect of both drugs at the specific concentrations. CI values marked in red are > 1, indicating antagonism. White bars show BRAFi treatment alone, grey bars show the tested kinase inhibitor alone and black bars represent the combined drugs. One representative experiment of at least 3 is shown. B) A375 cells were treated for 72 h with the indicated concentrations of MK-1775 (Wee1i), AZD7762 (Chki), Danusertib (Aurora kinase i) and TAE226 (FAKi) or CHIR-124 (Chki) in combination with either Vemurafenib (upper panel) or Dabrafenib (lower panel) and cell viability was assessed. The synergy score for each combination was calculated using the Synergyfinder software. Concentrations marked with green boxes on the x and y-axis indicate the concentrations encompassing the region of highest synergy (indicated by the white rectangle). The value in the white box represents the averaged score for the region of highest synergy. One representative experiment of at least three biological replicates is shown. (PDF 194 kb)

Additional file 6: **Figure S4.** Western blot analysis for selected drug treatments and apoptosis assays in healthy and melanoma cells. A) Western Blot analysis of A375, A375-XP and A375-GP cells treated with the BRAFi Vemurafenib (PLX), Chki AZD7762 (AZD), Wee1i MK-1775 (MK), FAKi TAE226 (TAE) or combinations thereof. Cells were treated for 3 h with indicated concentrations of inhibitors. Actin staining was used as loading control. B) The combination of MK-1775 and AZD7762 efficiently induced apoptosis in primary melanoma cells (M45), but not so much in healthy cells. Cells were treated for 72 h with the indicated concentrations of MK-1775 (Wee1i) or AZD7762 (Chki) or a combination thereof. Etoposide (Eto) treatment was used as positive apoptosis control. Resulting caspase-3 activity was normalized to the untreated control. 1 representative experiment out of 3 is shown. C) Western blot analysis of NHEM, NHDF and M45 primary melanoma cells after treatment for 3 or 24 h with indicated amounts of drugs. P-cdc2 (CDK1), cdc2 (CDK1), p-Chk1 and Chk1 were detected after 3 h drug treatment, while PARP cleavage was detected after 24 h treatment. Vinculin and α-tubulin were used as loading controls. AZD: AZD7762, MK: MK-1775; NHEM. Normal human epidermal melanocytes, NHDF: normal human dermal fibroblasts. (PDF 306 kb)

Additional file 7: **Figure S5.** Chou-Talalay analysis: A combination of Wee1 and Chk inhibitors synergistically inhibits proliferation of sensitive and resistant melanoma cells. A) Parental and BRAFi-resistant A375 cells were treated with MK-1775 (Wee1i) alone or in combination with either AZD7762 (left panel) or CHIR-124 (right panel) (both Chki). B) Parental and BRAFi-resistant IGR37 (left panel), 501Mel (right panel) cells and C) intrinsically resistant IGR39 cells were treated with MK-1775 (Wee1i) alone or in combination with AZD7762 (Chki). After 72 h, cell viability was determined. A dose-effect analysis of the drug combination was performed using the Compusyn software. CI values shown above the bars were mostly < 1 indicating a synergistic effect of both drugs at the specific concentrations. CI values marked in red are > 1, indicating antagonism. White bars show Wee1i (MK-1775) treatment alone, grey bars show Chki (AZD7762 or CHIR-124) treatment alone and black bars show the combined drugs. Red arrows pinpoint the most effective combinations. One representative experiment of at least 3 is shown here. A375/IGR37/501Mel-XP: resistant to Vemurafenib; A375/IGR37/501Mel-GP: resistant to Dabrafenib. (PDF 294 kb)

Additional file 8: **Figure 6.** Dose-response curves of selected kinase inhibitors in the presence or absence of BRAF inhibitors in healthy cells. A) NHEM and B) NHDF cells were subjected to 3-fold serial dilutions of each kinase inhibitor in the presence or absence of constant amounts of Vemurafenib (5 µM) or Dabrafenib (100 nM). Cell viability was assessed 72h after treatment by measuring cell viability. One representative curve of at least 3 biological replicates is depicted. NHEM: normal human epidermal melanocytes; NHDF: normal human dermal fibroblasts. (PDF 238 kb)

## Abbreviations

Aurk: Aurora kinase
Bcr-Abl: Breakpoint cluster region-Abelson
BRAF: B-Raf proto-oncogene serine/threonine kinase/v-raf murine sarcoma viral oncogene homolog B
CDK: Cyclin-dependent kinase
Chk: Checkpoint kinase
CI: Combination index
DNA-PK: DNA-activated protein kinase
FAK: Focal adhesion kinase
FGFR: Fibroblast growth factor receptor
GP: Dabrafenib/GSK2118436-resistant pool
HSA: Highest Single Agent
i: Inhibitor
IC_50_: Half-maximal inhibitor concentration
iRFP: Near-infrared fluorescent protein
MAPK: Mitogen activated protein kinase
MEK: MAPK/Erk kinase
NHDF: normal human dermal fibroblasts
NHEM: normal human epidermal melanocytes
NRAS: neuroblastoma RAS viral oncogene homolog
PFS: Progression-free survival
PI3K: Phosphoinositide 3-kinase
Plk: Polo-like kinase
RS: Replicative stress
wt: wild-type
XP: Vemurafenib/PLX4032-resistant pool
ZIP: Zero Interaction Potency
