# Supplementary material for: Kinase inhibitor library screening identifies synergistic drug combinations effective in sensitive and resistant melanoma cells
Source: J Exp Clin Cancer Res. 2019 Feb 6;38:56. doi: 10.1186/s13046-019-1038-x (PMC6364417; doi:10.1186/s13046-019-1038-x)
Supplement: Supplementary file 2 — Table S2. Mutation status of genes related to this study. Data are based on own sequencing experiments (WES and Sanger sequencing), online available data from the “COSMIC cell lines project” (https://cancer.sanger.ac.uk/cell_lines) and on literature (Halaban et al., Pigm Cell Mel Res, 2010). Synonymous mutations or mutations in non-coding sequences were not taken into account here. wt: no mutation detected; ni: no information available. Genomic profiles (exome sequencing) of the cell lines (A375, -XP and –GP, IGR37, -XP and –GP and IGR39) are available upon request. (PDF 63 kb) [file 13046_2019_1038_MOESM2_ESM.pdf]

| | A375 | A375-XP | A375-GP | IGR37 | IGR37-XP | IGR37-GP | IGR39 | 501Mel |
|---|---|---|---|---|---|---|---|---|
| **BRAF** | V600E | V600E | V600E | V600E | V600E | V600E | V600E | V600E |
| **NRAS** | wt | G13R | Q61K | wt | wt | wt | wt | wt |
| **NF1** | wt | wt | wt | wt | wt | wt | wt | ni |
| **PTEN** | wt | wt | wt | wt | wt | wt | wt | wt |
| **CDKN2A** | E69X E61X W66R | E69X E61X W66R | E69X E61X W66R | wt | wt | wt | wt | ni |
| **TP53** | wt | wt | wt | C229fs*10 | C229fs*11 | C229fs*12 | C229fs*12 | wt |
| **AURKB** | wt | wt | wt | F88L | F88L | F88L | F88L | ni |
| **AURKA** | I57V | I57V | I57V | I57V | I57V | I57V | I57V | ni |
| **CHEK1** | I471V | I471V | I471V | I471V | I471V | I471V | I471V | ni |
| **CHEK2** | wt | wt | wt | wt | wt | wt | wt | ni |
| **WEE1** | wt | wt | wt | A98T | A98T | A98T | A98T | ni |
| **PTK2 (FAK)** | wt | wt | wt | wt | wt | wt | wt | ni |
| **MAP2K1 (MEK)** | wt | wt | wt | wt | wt | wt | wt | ni |
| **PLK1** | wt | wt | wt | wt | wt | wt | wt | ni |
| **PRKDC (DNA-PK)** | wt | wt | wt | wt | wt | wt | wt | ni |
| **CDK1** | wt | wt | wt | wt | wt | wt | wt | ni |
| **PIK3CA** | wt | wt | wt | wt | wt | wt | wt | ni |
| **PIK3CB** | wt | wt | wt | wt | wt | wt | wt | ni |
| **PIK3CC** | wt | wt | wt | wt | wt | wt | wt | ni |
| **PIK3CD** | wt | wt | wt | wt | wt | wt | wt | ni |
| **PIK3R1** | wt | wt | wt | wt | wt | wt | wt | ni |

wt: no mutation detected

ni: no information
