# Supplementary figures and images for: Kinase inhibitor library screening identifies synergistic drug combinations effective in sensitive and resistant melanoma cells
Source: J Exp Clin Cancer Res. 2019 Feb 6;38:56. doi: 10.1186/s13046-019-1038-x (PMC6364417; doi:10.1186/s13046-019-1038-x)

Additional Figure 1

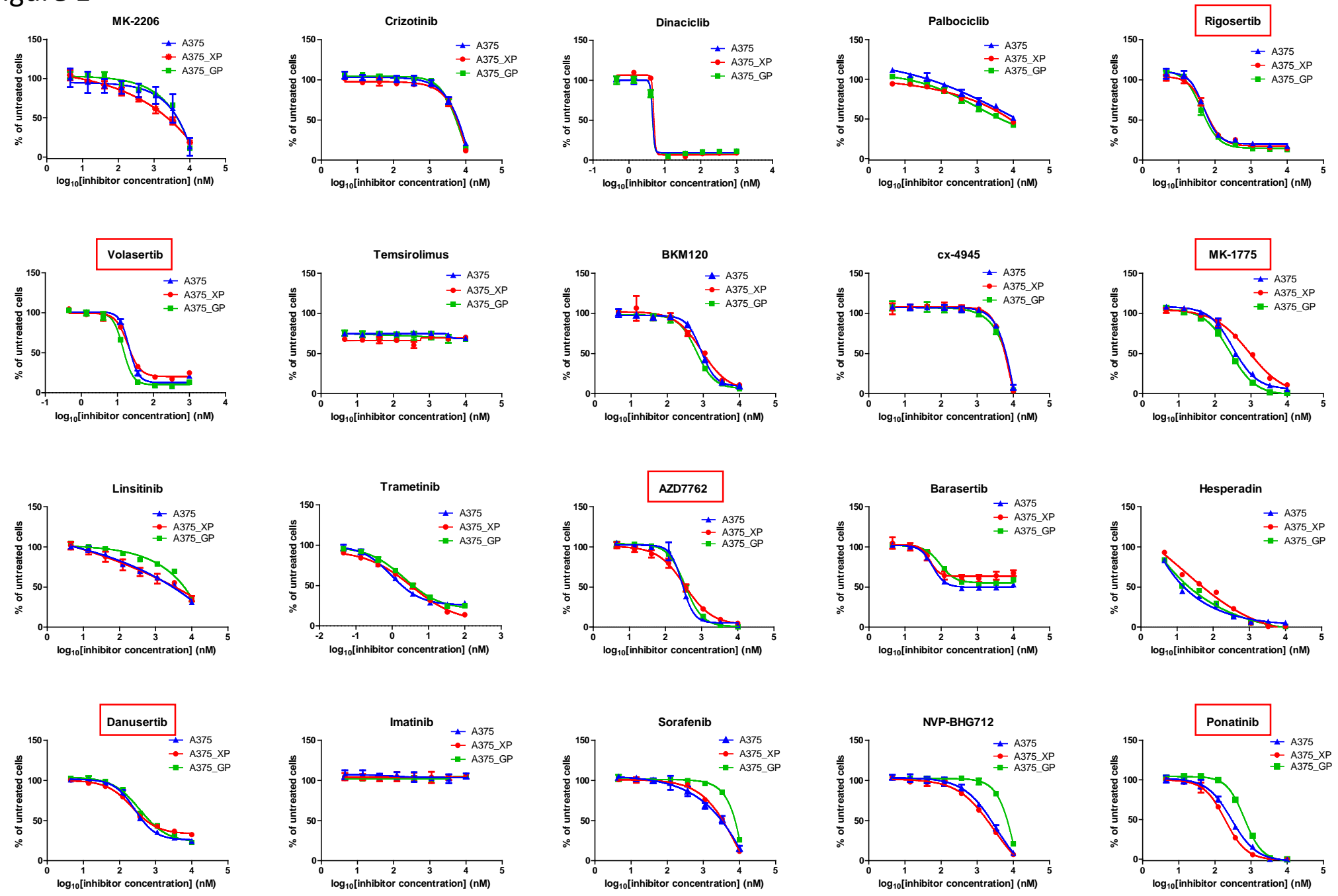

# Additional Figure 1, continued

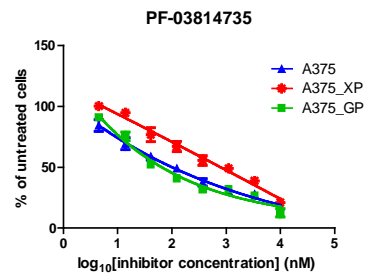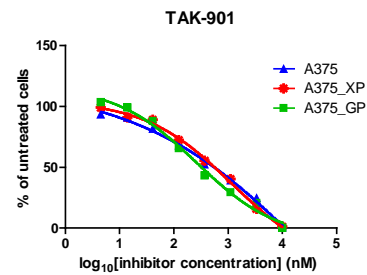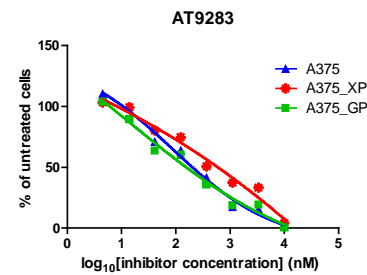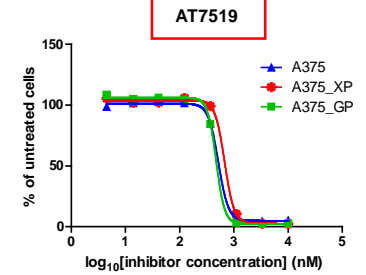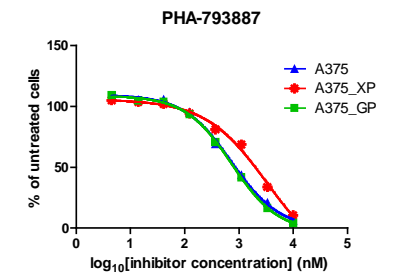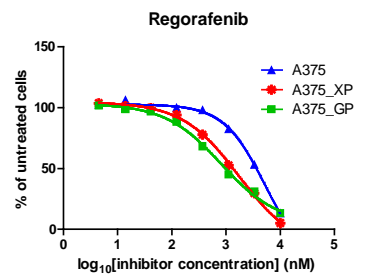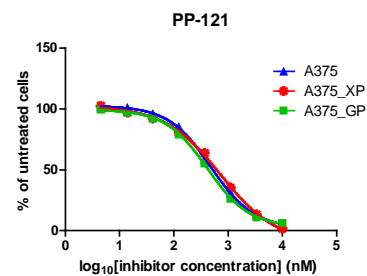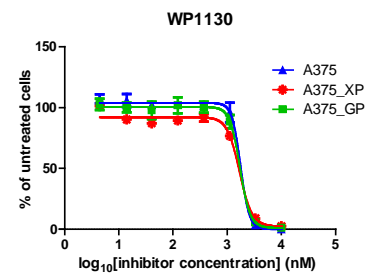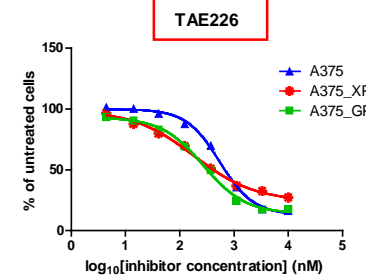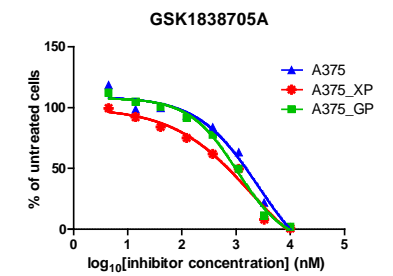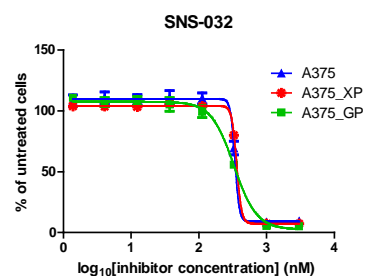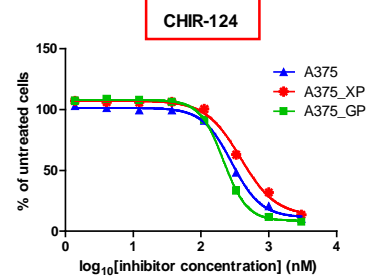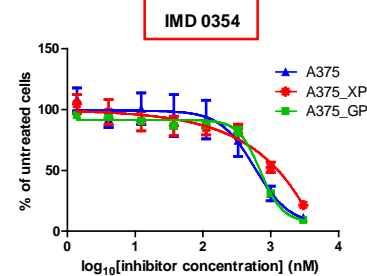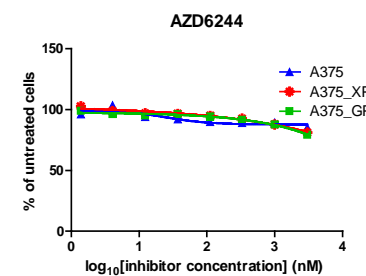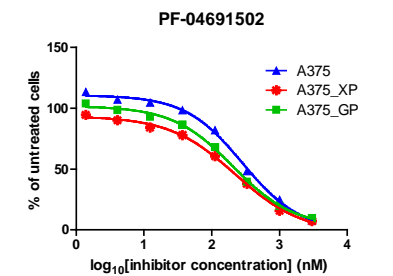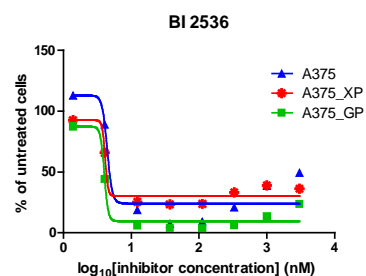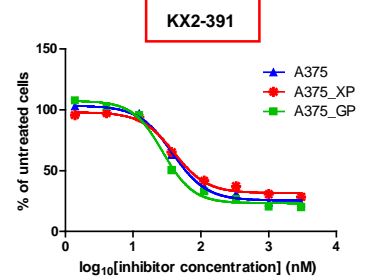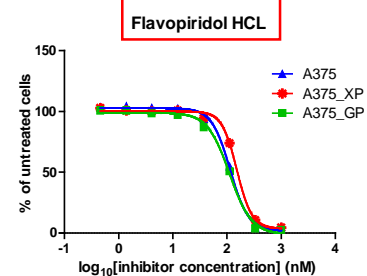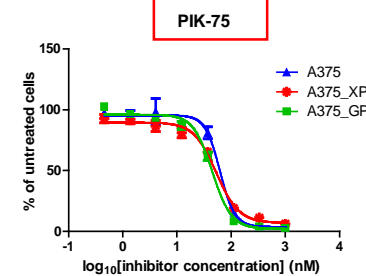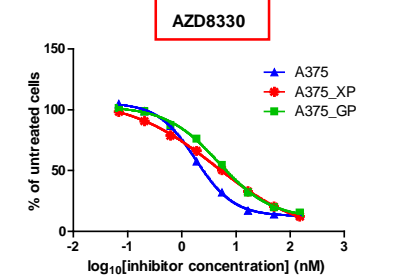

Supplement: Supplementary file 3 — Figure S1. Dose-response curves of selected kinase inhibitors in parental and BRAFi-resistant A375 cells. Response to 3-fold serial dilutions of each kinase inhibitor was assessed 72 h after treatment by measuring cell viability. Interesting candidates further tested in combination treatments in A375 cells are highlighted by a red frame (see also Table 1). One representative curve of at least 3 biological replicates is depicted here. _XP: cells resistant to Vemurafenib, _GP: cells resistant to Dabrafenib. (PDF 1030 kb) [file 13046_2019_1038_MOESM3_ESM.pdf]

Additional Figure 2

A

IGR37

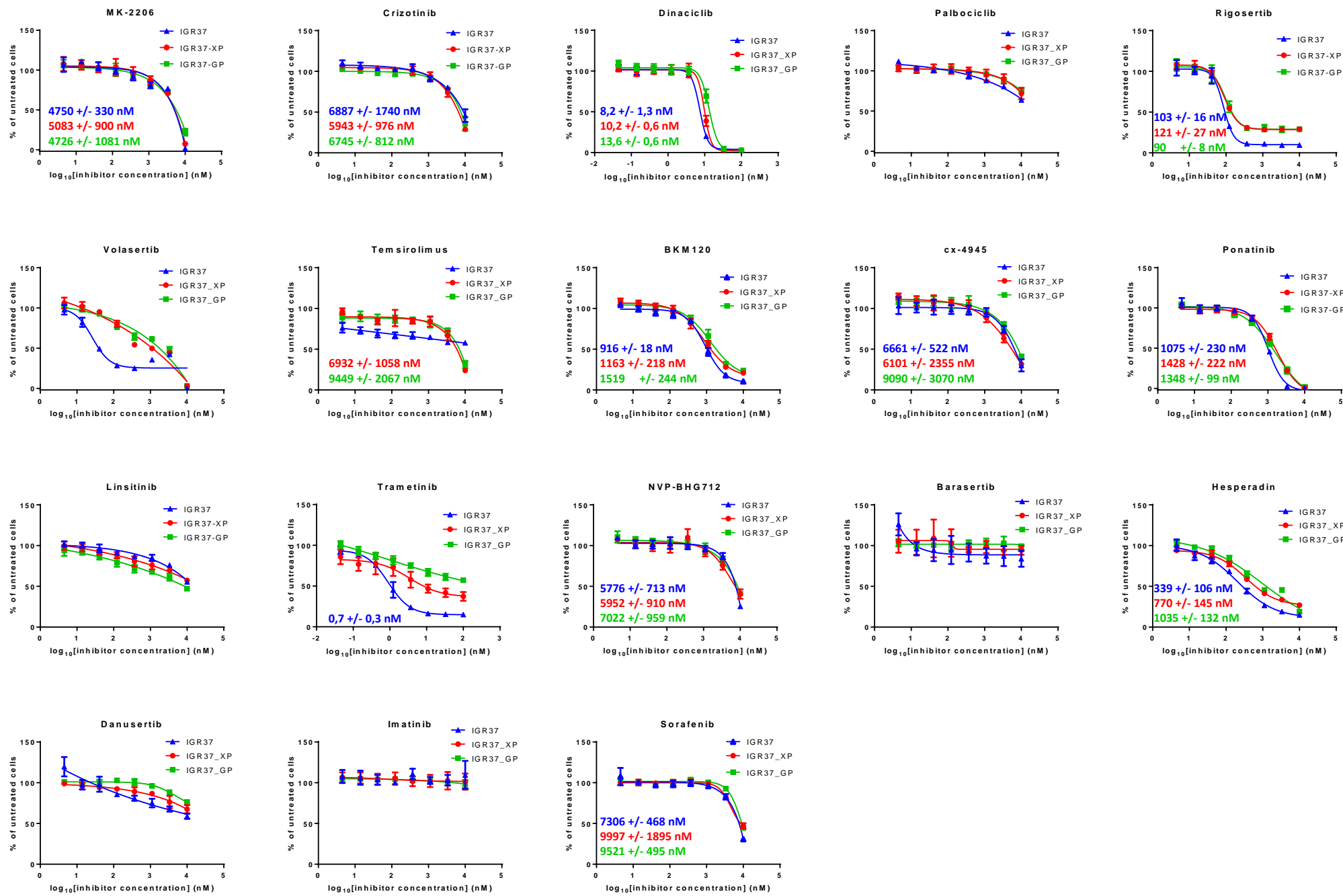

# Additional Figure 2

B

501Mel

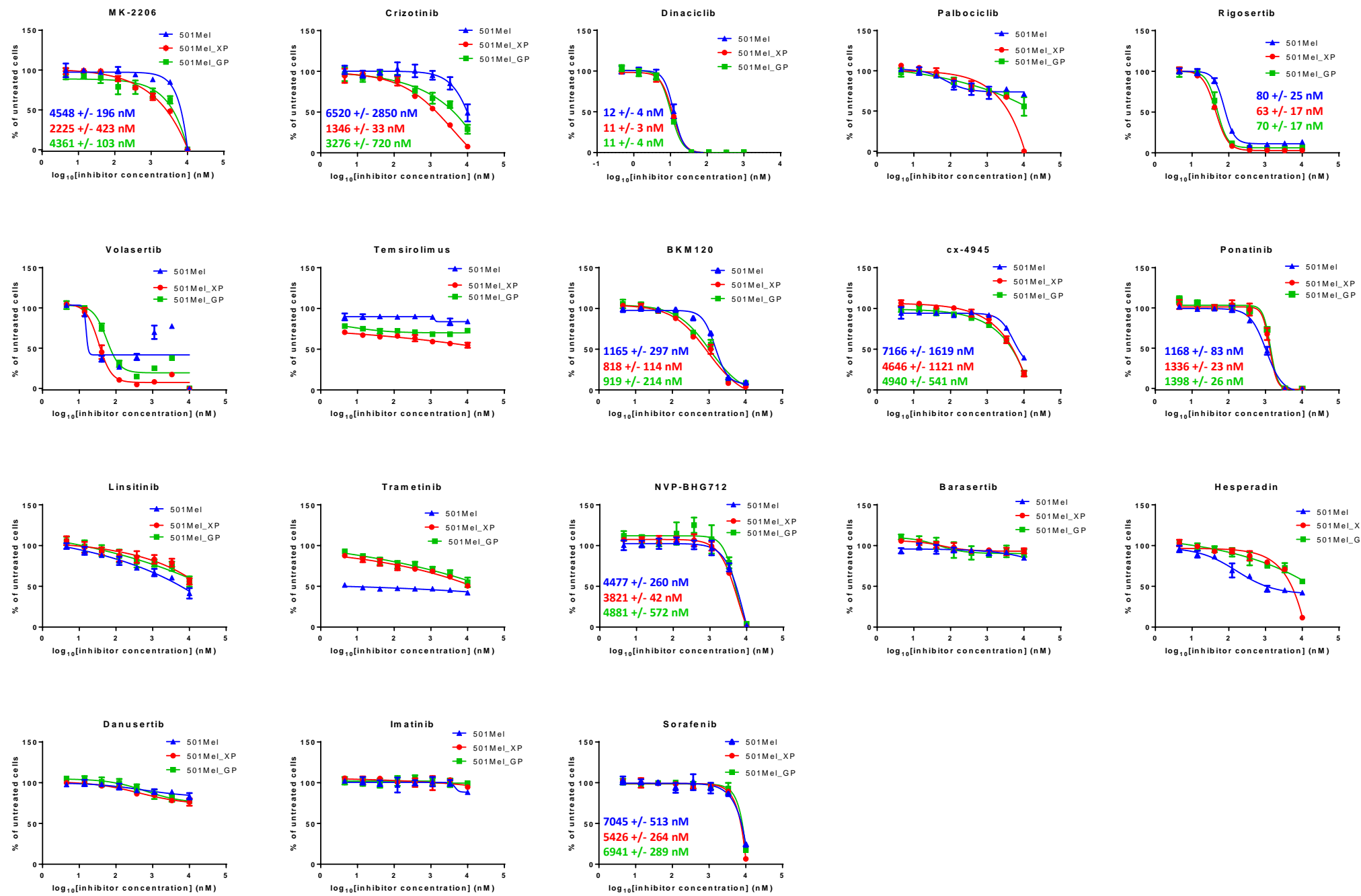

Supplement: Supplementary file 4 — Figure S2. Dose-response curves of selected kinase inhibitors in parental and BRAFi-resistant IGR37 and 501Mel cells. Response to 3-fold serial dilutions of each kinase inhibitor was assessed 72 h after treatment by measuring cell viability in IGR37 (A) and 501Mel (B) cells. The values depicted in the different graphs indicate the half-maximal inhibitory concentrations (IC50) of inhibitors for which IC50 values could be determined (as explained in Methods). Values represent the mean of at least three biological replicates; one representative curve of at least 3 biological replicates is depicted. _XP: cells resistant to Vemurafenib (red), _GP: cells resistant to Dabrafenib (green). (PDF 304 kb) [file 13046_2019_1038_MOESM4_ESM.pdf]

Additional Figure 3

A

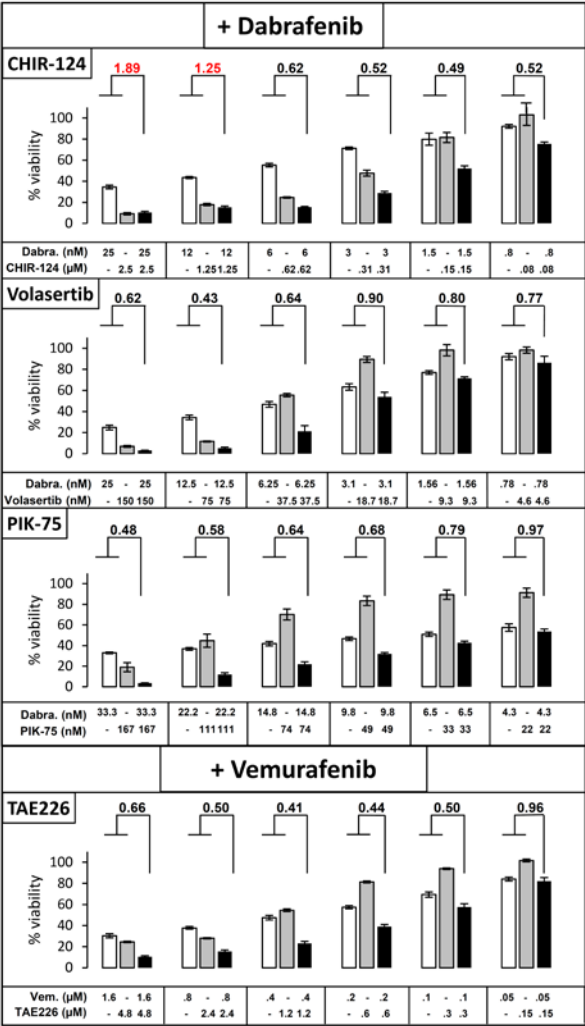

B

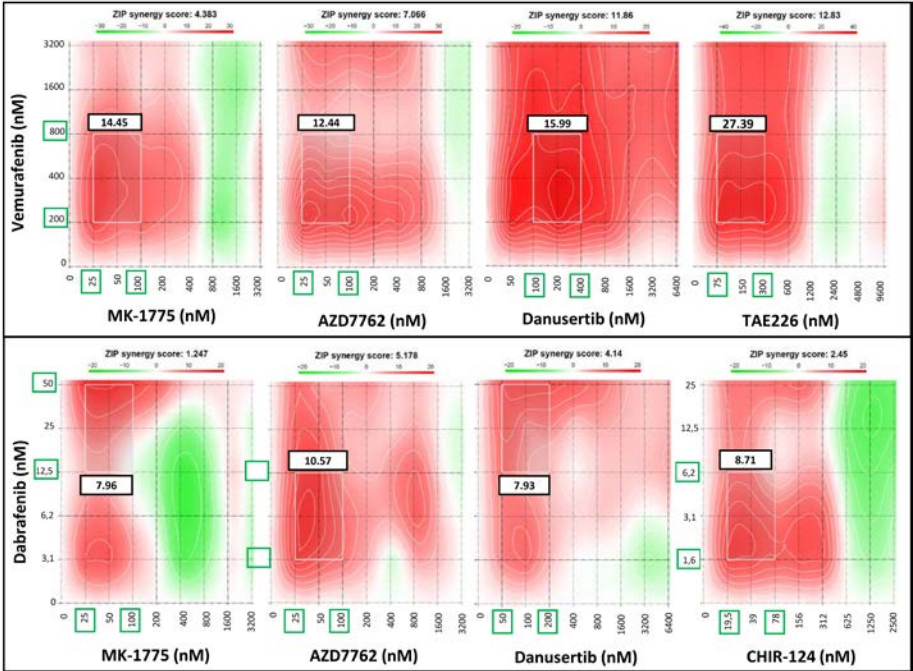

Supplement: Supplementary file 5 — Figure S3. BRAF inhibitors in combination with selected kinase inhibitors synergistically inhibit proliferation of A375 melanoma cells. A) A375 cells were treated for 72 h with Dabrafenib alone or in combination with CHIR-124 (Chki), Volasertib (Plki) or PIK-75 (PI3Ki, DNA-PKi), or with Vemurafenib alone or combined with TAE226 (FAKi) and cell viability was determined . A dose-effect analysis of the drug combination based on the Chou-Talalay method was performed using the Compusyn software. CI values shown above the bars were mostly < 1 indicating a synergistic effect of both drugs at the specific concentrations. CI values marked in red are > 1, indicating antagonism. White bars show BRAFi treatment alone, grey bars show the tested kinase inhibitor alone and black bars represent the combined drugs. One representative experiment of at least 3 is shown. B) A375 cells were treated for 72 h with the indicated concentrations of MK-1775 (Wee1i), AZD7762 (Chki), Danusertib (Aurora kinase i) and TAE226 (FAKi) or CHIR-124 (Chki) in combination with either Vemurafenib (upper panel) or Dabrafenib (lower panel) and cell viability was assessed. The synergy score for each combination was calculated using the Synergyfinder software. Concentrations marked with green boxes on the x and y-axis indicate the concentrations encompassing the region of highest synergy (indicated by the white rectangle). The value in the white box represents the averaged score for the region of highest synergy. One representative experiment of at least three biological replicates is shown. (PDF 194 kb) [file 13046_2019_1038_MOESM5_ESM.pdf]

Additional Figure 4

A

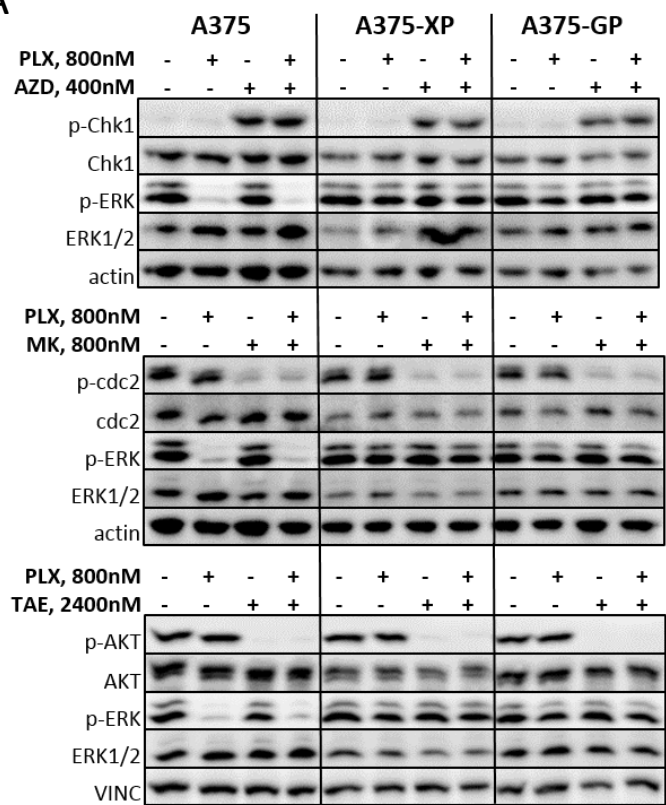

B

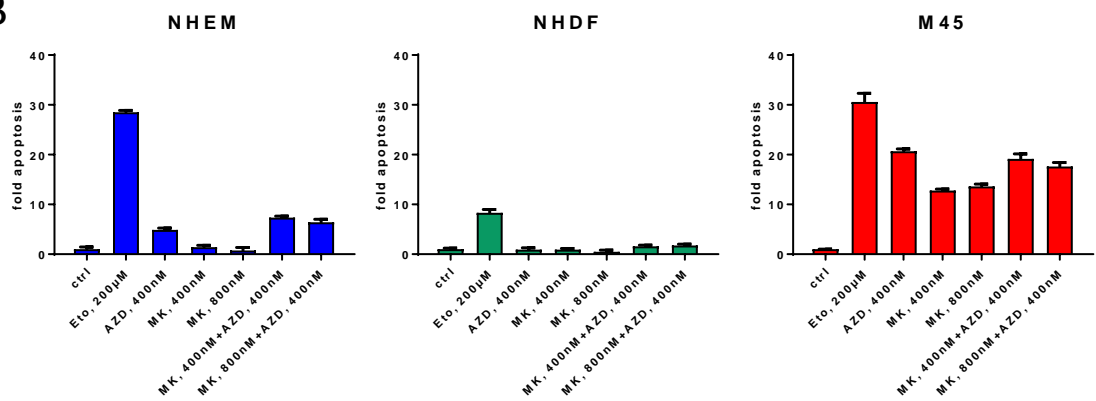

C

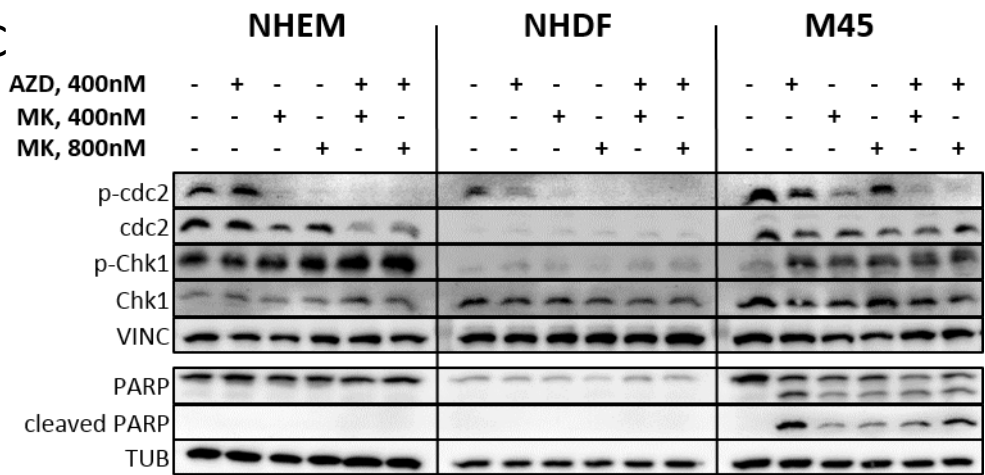

Supplement: Supplementary file 6 — Figure S4. Western blot analysis for selected drug treatments and apoptosis assays in healthy and melanoma cells. A) Western Blot analysis of A375, A375-XP and A375-GP cells treated with the BRAFi Vemurafenib (PLX), Chki AZD7762 (AZD), Wee1i MK-1775 (MK), FAKi TAE226 (TAE) or combinations thereof. Cells were treated for 3 h with indicated concentrations of inhibitors. Actin staining was used as loading control. B) The combination of MK-1775 and AZD7762 efficiently induced apoptosis in primary melanoma cells (M45), but not so much in healthy cells. Cells were treated for 72 h with the indicated concentrations of MK-1775 (Wee1i) or AZD7762 (Chki) or a combination thereof. Etoposide (Eto) treatment was used as positive apoptosis control. Resulting caspase-3 activity was normalized to the untreated control. 1 representative experiment out of 3 is shown. C) Western blot analysis of NHEM, NHDF and M45 primary melanoma cells after treatment for 3 or 24 h with indicated amounts of drugs. P-cdc2 (CDK1), cdc2 (CDK1), p-Chk1 and Chk1 were detected after 3 h drug treatment, while PARP cleavage was detected after 24 h treatment. Vinculin and α-tubulin were used as loading controls. AZD: AZD7762, MK: MK-1775; NHEM. Normal human epidermal melanocytes, NHDF: normal human dermal fibroblasts. (PDF 306 kb) [file 13046_2019_1038_MOESM6_ESM.pdf]

Additional Figure 5

A

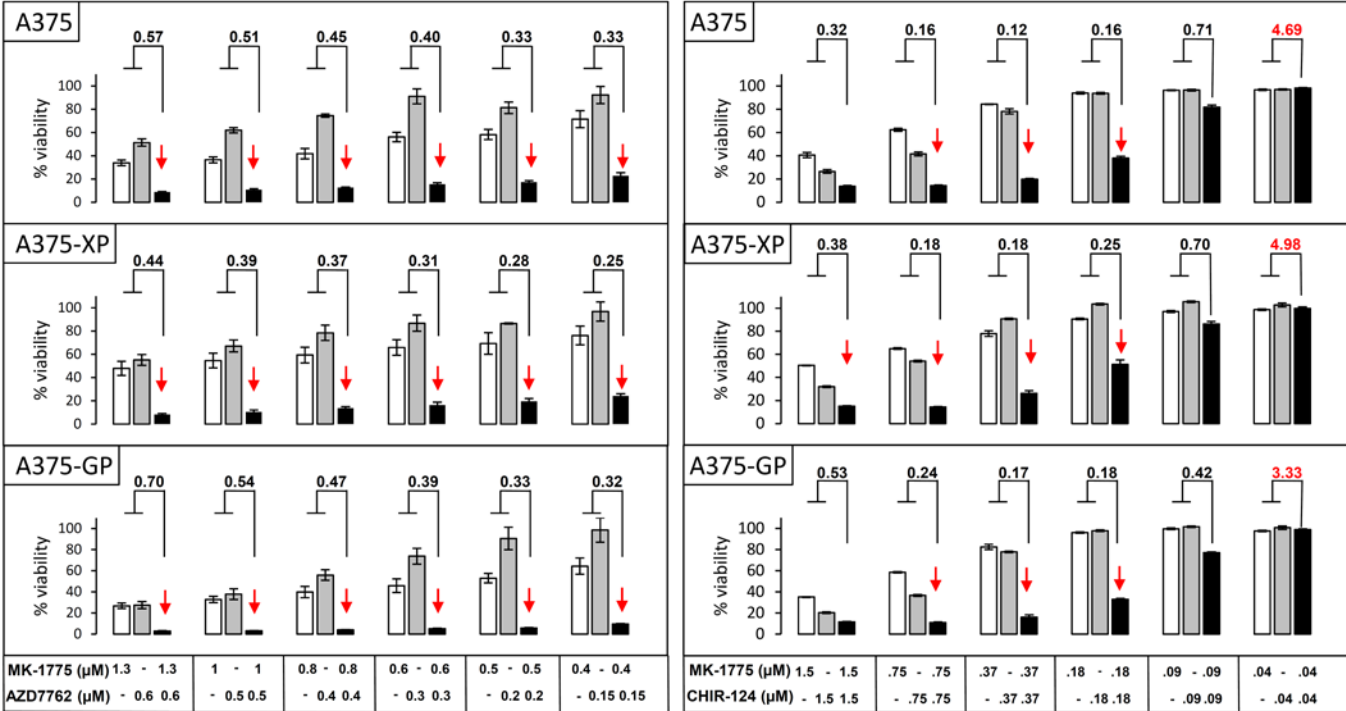

B

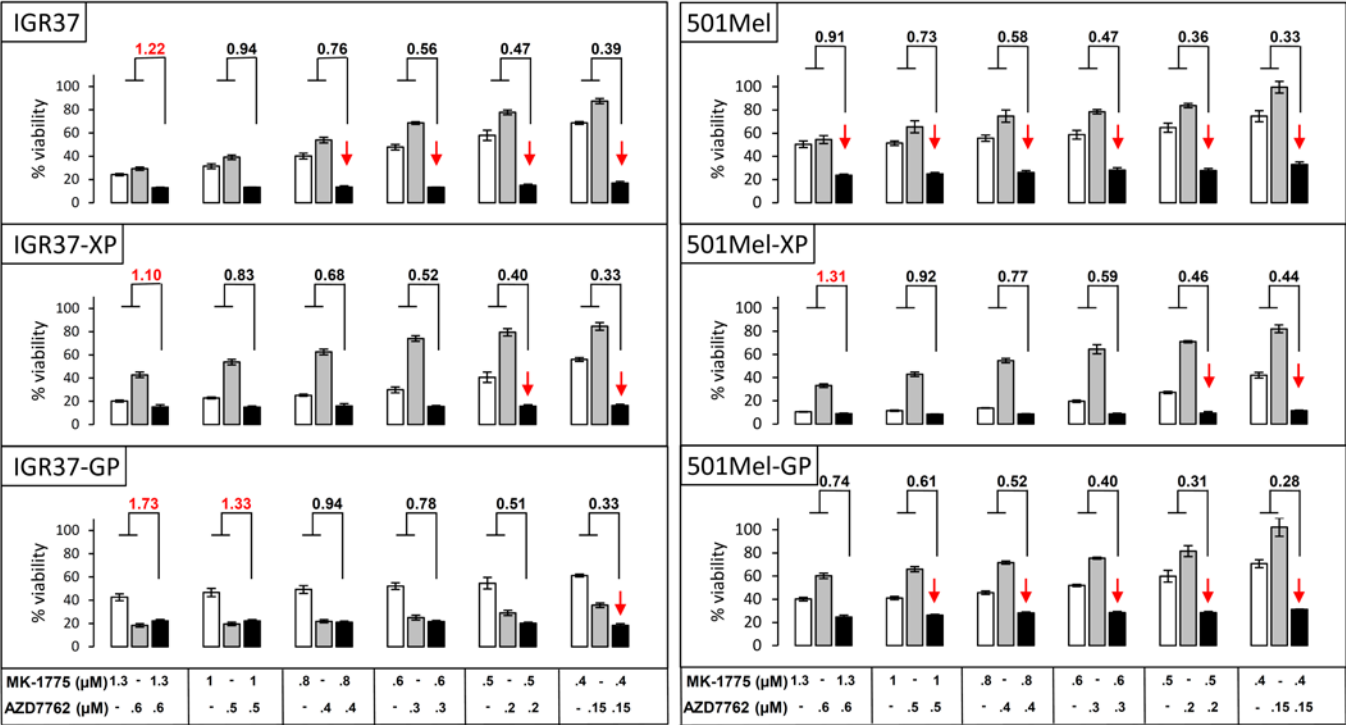

C

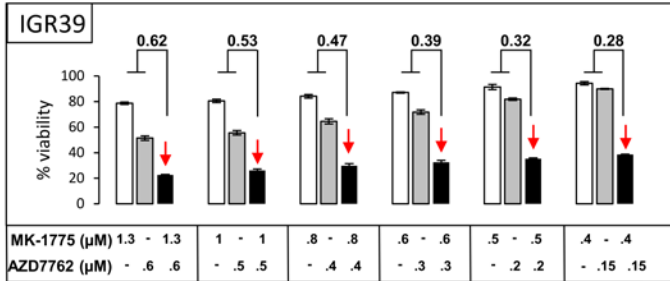

Supplement: Supplementary file 7 — Figure S5. Chou-Talalay analysis: A combination of Wee1 and Chk inhibitors synergistically inhibits proliferation of sensitive and resistant melanoma cells. A) Parental and BRAFi-resistant A375 cells were treated with MK-1775 (Wee1i) alone or in combination with either AZD7762 (left panel) or CHIR-124 (right panel) (both Chki). B) Parental and BRAFi-resistant IGR37 (left panel), 501Mel (right panel) cells and C) intrinsically resistant IGR39 cells were treated with MK-1775 (Wee1i) alone or in combination with AZD7762 (Chki). After 72 h, cell viability was determined. A dose-effect analysis of the drug combination was performed using the Compusyn software. CI values shown above the bars were mostly < 1 indicating a synergistic effect of both drugs at the specific concentrations. CI values marked in red are > 1, indicating antagonism. White bars show Wee1i (MK-1775) treatment alone, grey bars show Chki (AZD7762 or CHIR-124) treatment alone and black bars show the combined drugs. Red arrows pinpoint the most effective combinations. One representative experiment of at least 3 is shown here. A375/IGR37/501Mel-XP: resistant to Vemurafenib; A375/IGR37/501Mel-GP: resistant to Dabrafenib. (PDF 294 kb) [file 13046_2019_1038_MOESM7_ESM.pdf]

Additional Figure 6

A

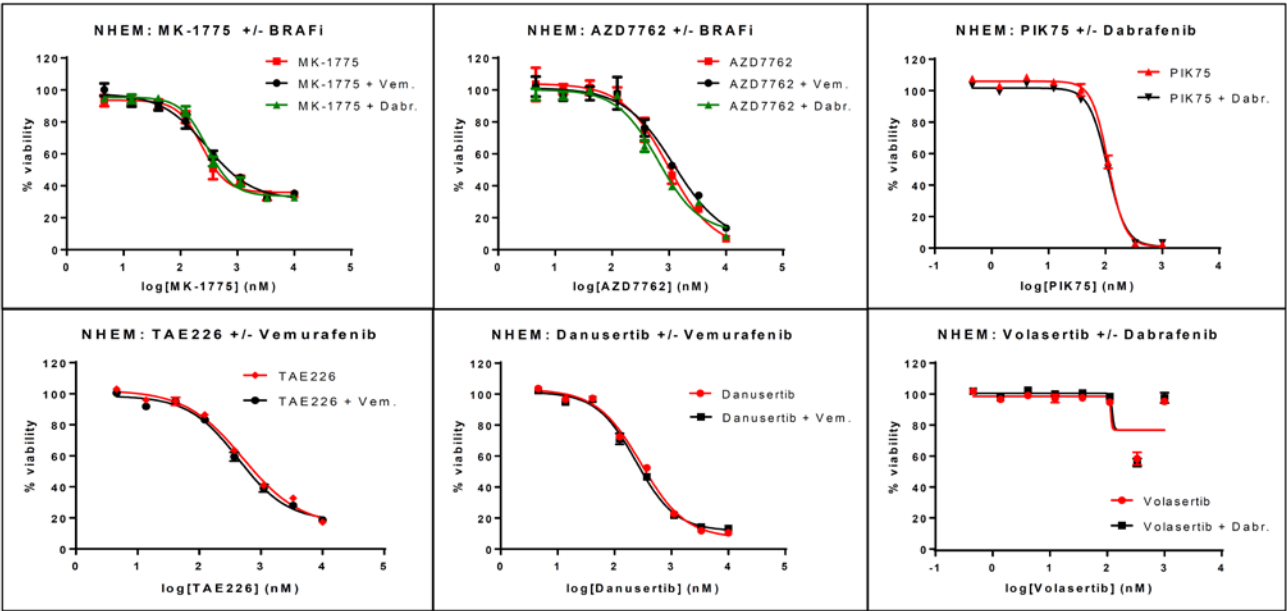

B

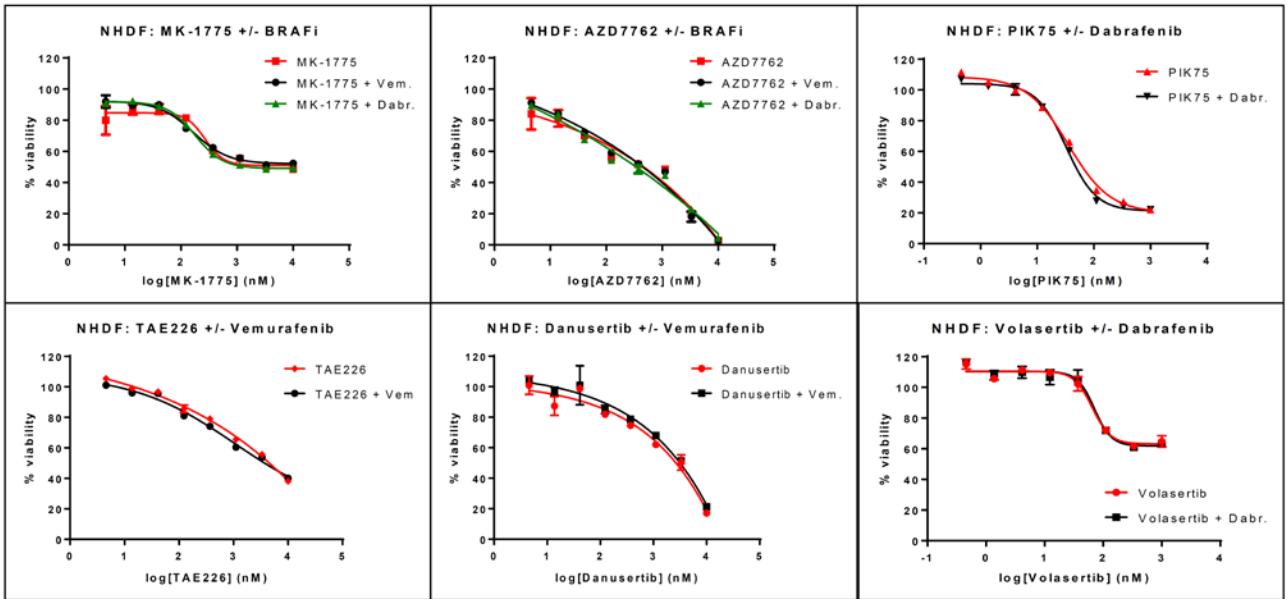

Supplement: Supplementary file 8 — Figure 6. Dose-response curves of selected kinase inhibitors in the presence or absence of BRAF inhibitors in healthy cells. A) NHEM and B) NHDF cells were subjected to 3-fold serial dilutions of each kinase inhibitor in the presence or absence of constant amounts of Vemurafenib (5 µM) or Dabrafenib (100 nM). Cell viability was assessed 72h after treatment by measuring cell viability. One representative curve of at least 3 biological replicates is depicted. NHEM: normal human epidermal melanocytes; NHDF: normal human dermal fibroblasts. (PDF 238 kb) [file 13046_2019_1038_MOESM8_ESM.pdf]
